# Spatio–temporal dynamics of bacterial community composition in a Western European watershed, the Meuse River watershed

**DOI:** 10.1093/femsec/fiaf022

**Published:** 2025-03-05

**Authors:** Valentin Barberoux, Adriana Anzil, Loïc Meinertzhagen, Thanh Nguyen-Dinh, Pierre Servais, Isabelle F George

**Affiliations:** Laboratory of Ecology of Aquatic Systems (ESA), Brussels Bioengineering School, Université Libre de Bruxelles, Brussels 1050, Belgium; Laboratory of Marine Biology, Faculty of Sciences, Université Libre de Bruxelles, Brussels 1050, Belgium; Laboratory of Ecology of Aquatic Systems (ESA), Brussels Bioengineering School, Université Libre de Bruxelles, Brussels 1050, Belgium; Laboratory of Ecology of Aquatic Systems (ESA), Brussels Bioengineering School, Université Libre de Bruxelles, Brussels 1050, Belgium; Greening Laboratory, Biomedicine Discovery Institute, Monash University, Victoria 3800, Australia; Laboratory of Ecology of Aquatic Systems (ESA), Brussels Bioengineering School, Université Libre de Bruxelles, Brussels 1050, Belgium; Laboratory of Ecology of Aquatic Systems (ESA), Brussels Bioengineering School, Université Libre de Bruxelles, Brussels 1050, Belgium

**Keywords:** bacterial community composition, bioindicator, fraction size, headwater, Meuse River, watershed

## Abstract

This study aimed to identify factors influencing bacterial diversity in the Meuse River watershed by analyzing 42 locations sampled in spring and summer 2019, combined with biweekly sampling of one mid-stream location for a year. Bacterial community composition (BCC) was assessed in the small (SF; <5 µm) and large fractions (LF; ≥5 µm,), alongside physico–chemical parameters. LF consistently exhibited greater alpha diversity than SF. During the spatial campaigns, alpha diversity increased downstream in spring with high discharge, and BCC differed significantly between headwaters and the main river. Along this axis, several genera, *Flavobacterium, Limnohabitans*, and *Aquirufa* stood out as indicators of good water quality. *Rhodoferax*, another taxon indicative of good water quality, prevailed in the headwaters and during winter. In contrast, two cyanobacteria genera indicators of poor river quality, *Microcystis PCC 7914* and *Cyanobium PCC 6307*, peaked in summer. BCC in spring and summer temporal samples aligned with spatial ones, while winter and autumn samples had distinct BCC. Finally, season, temperature, and distance from river mouth were the main driving parameters of beta diversity, outweighing the effect of fraction size on the BCC. These findings reinforce the notion that local conditions exert significant influence on bacterial communities in rivers.

## Teaser

This study reveals how seasonal changes, temperature, and location shape bacterial diversity in the Meuse River watershed, highlighting specific bacterial indicators of water quality across different river sections.

## Introduction

Bacteria are integral to river ecosystems, where they contribute to vital biogeochemical processes such as organic matter decomposition and nitrification. Their importance is illustrated by the microbial loop, i.e. the assimilation of dissolved organic matter into biomass by bacteria, which are then ingested by protists, themselves predated by zooplankton (Azam et al. 1983). This pathway of carbon and nutrient cycling though microbial components is coupled to the classic food chain formed by the phytoplankton–zooplankton–fish hierarchy.

Like in other environments, the analysis of bacterial community composition (BCC) in rivers has benefited from the rapid evolution of biomolecular techniques that started with low-resolution fingerprinting followed by next-generation sequencing and more recently metagenomics. It has been shown in many studies that river microbial assemblages are dominated by a limited number of phyla: *Actinomycetota, Pseudomonadota, Bacteroidota*, and *Cyanobacteriota* (previously named as *Actinobacteria, Proteobacteria, Bacteroidetes*, and *Cyanobacteria*, respectively) (Staley et al. 2013, de Oliveira and Margis 2015, Savio et al. 2015, Wang et al. 2016, 2019, Hu et al. 2017, Hassell et al. 2018, Henson et al. 2018, Blais et al. 2022). In particular, several genera have been frequently associated with freshwater environments, such as *hgcI clade* (*Actinomycetota*) (Kang et al. 2017, Newton et al. 2007), *Flavobacterium* (*Bacteroidota*) (Hagberg et al. 2021, Kirchman 2002), *Limnohabitans* (*Pseudomonadota*) (Kasalicky et al. 2013, Hu et al. 2018), and *Fluviicola* (*Bacteroidota*) (Guo et al. 2020, Ji et al. 2018).

One way to differentiate subgroups in aquatic bacterial communities is to analyze the BCC of particle-attached communities versus free-living ones. Indeed the water column is a heterogenous environment, where mineral or organic particles (e.g. flocs of decaying phytoplankton) provide various habitats and/or carbon sources for bacteria, therefore being considered as hotspots of microbial abundances and activity compared to free-living bacteria (Crump et al. 1998, Luef et al. 2007). Accordingly, many studies reported that river bacterial diversity is higher in the fraction recovered on 3- or 5-µm-sized filters (“particle-associated” bacteria) than in the flow through (“free-living” bacteria) (Crump et al. 1999, Velimirov et al. 2011, Savio et al. 2015, Payne et al. 2017, Henson et al. 2018, Liu et al. 2019).

Along the river course, variations in BCC have been reported, that are reminiscent of what is observed for benthic invertebrates and has been framed as the River Continuum Concept (Vannote et al. 1980). This concept describes how the physico–chemical characteristics of a river change along its course, leading to a predictable succession of biological communities. In several studies, such a succession has been observed for bacterial communities (Staley et al. 2013, Savio et al. 2015). Headwaters (HW) hold a diverse community of little active soil- and groundwater-affiliated taxa (Crump et al. 2012, Savio et al. 2015) or, on the contrary, fast-growing r-strategists (e.g. some *Bacteroidota*) (Read et al. 2015). Then, as the river progresses, the latter are progressively replaced by “typical” k-strategists (Read et al. 2015), which are small, nonmotile, slow-growing substrate specialists (Savio et al. 2015, Niño-García 2016) belonging, among others, to the *hgcI* I clade (*Actinomycetota*), and the *Polynucleobacter* and *Limnohabitans* genera (both *Pseudomonadota*) (Livermore et al. 2014, Pernthaler 2017). Generally speaking, the structure of biological communities is described in the literature as the result of the interplay of two antagonistic mechanisms: “mass effect” and “species sorting” (Mouquet and Loreau 2002, 2003, Cadotte 2006, Shanafelt et al. 2015, Thompson and Gonzalez 2016, Leibold et al. 2017). For riverine bacterial communities, the “mass effect” process can be portrayed as the input of allochthonous bacteria originating from surrounding riparian zone that, when prevailing, leads to higher alpha diversity and lower beta diversity and the dominance of certain species such as those typical of soils (Wang et al. 2019). This phenomenon holds particular significance in HW ecosystems. Those species are not the most locally adapted ones, but they are the most abundant ones at a regional scale. Conversely, “species sorting” is the selection of the most fit species by the local (a)biotic parameters, leading to a lower alpha diversity and a higher beta diversity across the rivers of a watershed (Suzuki and Economo 2021).

In various studies, a gradual shift from mass effect to species sorting has been described along the river course. Indeed, as bacteria flow downstream, they face increased competition for resources (Crump et al. 2012, Savio et al. 2015, Niño-García et al. 2016), favoring the proliferation of the most competitive species. Conversely, other studies reported a stability in terms of alpha diversity along the river course (Staley et al. 2014, Wang et al. 2019) or an increase downriver (Henson et al. 2018), with no clear shift in terms of beta diversity. This variety of results between different studies suggest that the balance between local and regional processes differs from one river ecosystem to another.

In addition, the specific parameters influencing BCC appear to differ considerably between river watersheds, thus hindering the identification of universally consistent factors. These identified driving parameters are either temperature (Ma et al. 2016, Reza et al. 2018, Cruaud et al. 2020, Wang et al. 2023), dissolved oxygen (DO; Feng et al. 2012, Spietz et al. 2015), pH (Niño-García et al. 2016, Doherty et al. 2017, Mateus-Barros et al. 2021), salinity (Ma et al. 2016), total suspended matter (TSM) (Sommaruga and Casamayor 2009), concentration and/or quality of organic matter (Judd et al. 2006, Staley et al. 2014), and nitrogen and/or phosphorus concentrations (Ma et al. 2016, Hu et al. 2020, Mateus-Barros et al. 2021). The impact of watershed characteristics has been highlighted as well, such as the distance from the river source (Paudel Adhikari et al. 2019), river discharge (Doherty et al. 2017, Cruaud et al. 2020, Caillon et al. 2021), landform (Liu et al. 2018), and land use or land cover (Van Rossum et al. 2015, Hosen et al. 2017). Some studies have also confirmed the influence of season, which integrates several abovementioned parameters, on BCC (Crump et al. 2009, Doherty et al. 2017). Lastly, the effect of biotic factors such as phytoplanktonic blooms (Winter et al. 2007) and composition (Šimek et al. 2011) or grazing rate by protozoa (Salcher et al. 2015) has been reported as well.

One step further, several studies have underscored the potential of bacteria as indicators of water quality due to their high sensitivity to variations in water physico–chemical parameters (Zhang et al. 2008, Martinez-Santos et al. 2018). Their effectiveness as proxies for ecological status has been demonstrated across various aquatic environments. For example, in coastal ecosystems, Aylagas et al. (2017) developed a bacterial biotic index that showed a significant correlation with anthropogenic compounds such as Polychlorinated Biphenyls (PCB), cadmium, and organic matter. Several families, among which *Comamonadaceae* and *Flavobacteriaceae*, were identified as indicators of poor ecological status. In the Songhua River, Yang et al. (2019) found that bacterial indicators of remediation could be identified based on their negative correlation with nitrate levels, including members of the *Comamonadaceae* family, *Limnohabitans, Flavobacterium*, and *Rhodoferax*. In the Danube River, Fontaine et al. (2023) utilized the negative correlation between bacterial taxa and Chl-a concentration—a proxy for eutrophication—to identify four genera as reliable indicators of good water quality: *Fluviicola, Acinetobacter, Flavobacterium, and Rhodoluna*.

**Figure 1.**
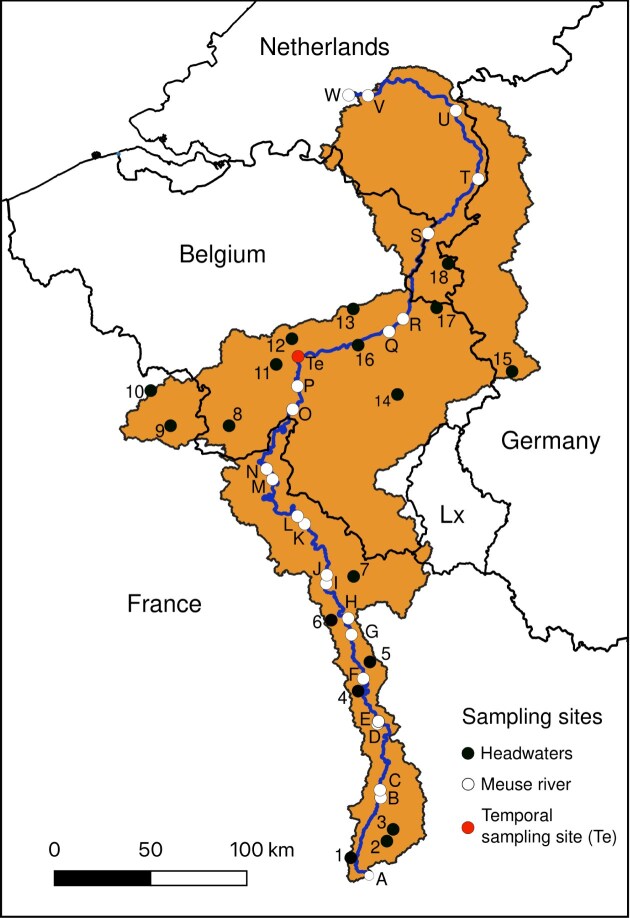
Map of the Meuse River watershed (in orange - contrasting with the background) and the sampling sites—Lx = Luxemburg..

The Meuse River, which is the focus of this study, is 926 km long, ranking as the 11th longest river of Western Europe, and crosses three countries (Belgium, France, and the Netherlands) (Fig. 1). Its watershed area is 34 548 km^2^ and is populated by roughly 7 millions inhabitants [2009 census in Descy (2009)], covering some parts of Germany too. Its annual discharge at Jambes (located midstream of the river) is 159 m^3^/s (hydrometrie.wallonie.be). Its water serves different purposes such as agriculture, industries, drinking water supply, hydroelectricity production, and recreational activities (Descy 2009). Since the 1980s, several surveys have been undertaken on this river centered on phytoplankton production (Descy 2002), bacterioplankton biomass and production (Servais 1989), planktonic food webs (Joaquim-Justo et al. 2006, Servais et al. 2000), dissolved carbon dioxide, methane and nitrous oxide concentrations (Borges 2018), or effect of floods on TSM (Hamers et al. 2015).

To our knowledge, this study provides the first comprehensive analysis of bacterial diversity in the Meuse River watershed, with a distinction between large and small fractions (SF). Three distinct sampling campaigns were undertaken: two spatial campaigns covering the entire watershed during spring and summer, when microbial activity is expected to peak, and one temporal campaign spanning a full year at a midstream site.

The main objective of this study was to investigate the evolution of the BCC within the Meuse watershed using a spatio–seasonal approach, where two fractions in the water column are considered separately (small versus large fraction). Specifically, this study aimed to: (i) assess whether spatial patterns of alpha diversity aligned with temporal ones, (ii) determine the extent to which environmental parameters influenced beta diversity, (iii) evaluate if the BCC of the Meuse watershed was dominated by typical freshwater taxa, and (iv) determine whether dominant taxa could serve as bioindicators of river quality based on their correlation with environmental factors.

## Materials and methods

### Study sites and sampling strategy

During the spatial campaigns, which took place during the spring and summer of 2019, 42 sampling sites were analyzed (Fig. 1). Twenty-four sites were sampled along the Meuse main river axis (MR) from the river source to its mouth, with a distance of roughly 30 km between sampling sites. Due to practical limitations, no sample was taken between the river spring and 69 km downstream.

Eighteen sites were sampled in HW, which were located within an area characterized by a single soil occupation and at maximum 5 km from a stream source. The QGIS 3.16.7 software was used to visualize maps of the Meuse watershed and its land use to choose the sampling stations. The area where the Meuse meets the Rhine and forms a delta was excluded from the watershed representation (Fig. 1) due to the complex water mixing in that section, which made it difficult to analyze the evolution of the Meuse BCC. Consequently, the study focused on a stretch of the river from its source to 926 km downstream, which corresponds to the entry point into the delta, with this point referred to as the “river mouth.” The temporal campaign was conducted at a site located midstream (Jambes, 440 km from the river mouth), which was sampled every second week for 1 year, from February 2019 to March 2020. GPS coordinates of all study sites can be found in Table S1, with sampling dates and values of studied parameters.

### Sample collection and analysis of (a)biotic parameters

In small streams, surface water was carefully sampled within the first 30 cm depth with a 10-l bucket. Elsewhere, where the river depth was superior to 1 m, surface water was collected using a bucket attached to a rope, from the middle of a bridge. Before collecting water, buckets were rinsed several times with the water from the same sampling site. Afterwards, water was transferred into 10-l bottles, which were rinsed the same way.

The experimental protocols are detailed in the Supplementary material. On site, temperature and DO were measured. In addition, for bacterial production measurement, 10 ml of water were poured in 50-ml plastic flasks and those were stored in boxes filled with river water in order to maintain the temperature close to the river one before incubation of the samples with the radioactive substrate (tritiated thymidine), which was performed at the laboratory. Other parameters were measured in the laboratory the same day: TSM, chemical oxygen demand (COD), and chlorophyll *a* (Chl-a). Phosphate and ammonium concentrations were measured for the samples of the spatial campaigns (HW and MR samples), but not for those of the temporal campaign due to logistical limitations. River discharge data were obtained from public institutions monitoring rivers in France (hydro.eaufrance.fr), Belgium (voies-hydrauliques.wallonie.be), and the Netherlands (rijkswaterstaat.nl). Those could not be determined for HW streams.

### DNA extraction, PCR, and sequencing

Water samples were first filtered on 5-µm pore-sized polycarbonate filters (Durapore, Merck Millipore, Ireland) to collect the “particle associated” bacteria, or large fraction bacteria (LF). Then the flow through was filtered on 0.2-µm pore-sized filters to collect “free living” bacteria, or SF bacteria. Filtration was performed until the filter was clogged, which typically occurred after ~1 l was filtered on a 5-µm filter and 300 ml on a 0.2-µm filter. DNA was extracted from the material retained on the membranes using a phenol–chloroform–isoamyl-based extraction protocol (detailed in the Supplementary materials).

A two-step Polymerase Chain Reaction (PCR) procedure was performed. PCR1 consisted in the 16 rRNA gene amplification and was executed in our laboratory, followed by gel electrophoresis to assess the quality of amplicons that were then stored at −20°C.

The amplification protocol and the primers used [515F (GTGYCAGCMGCCGCGGTAA) and 806 Rb (GGACTACNVGGGTWTCTAAT)] (Apprill et al. 2015, Parada et al. 2016) were those recommended by the Earth Microbiome Project to amplify the V4 region of the 16S rRNA gene (Caporaso et al. 2011, 2012). 2.5 µl of DNA (5 ng/µl) were put into a 0.2-ml PCR tube, with 5 µl of primer F (1 µM) and 5 µl of primer R (1 µM). Then, 12.5 µl of PCR mix were added (KAPA Hifi HotStart ReadyMix PCR Kit, Kapa Biosystems, Roche Sequencing, Switzerland). PCR1 was run as follows: 3 min at 95°C, 25 cycles of 30 s at 95°C, 30 s at 55°C, 30 s at 72°C, and a last step of 5 min at 72°C. Amplicons were stored at −20°C. PCR2 consisted in ligating the indexed adaptors to the amplicons. It was performed at the “Genotoul bioinformatics platform Toulouse Occitanie” (https://bioinfo.genotoul.fr), which also carried out the Illumina Mi-Seq paired-end sequencing (2 × 250 bp).

The sequences obtained were submitted to the NCBI Nucleotide Sequence Database (accession number: PRJNA1126447). SRA accession numbers are provided in Table S2.

### Bioinformatic pipeline and downstream analyses

The demultiplexed raw file of sequence data of the 205 samples was processed using the DADA2 pipeline v1.16 (Callahan et al. 2016) on the R v3.1 software (RStudio Team 2020). The process followed by this pipeline has already been detailed in a previous study (Fontaine et al. 2023). First, primers were removed, then dereplication, denoising, and concatenation of paired sequences were performed. Additionally, forward and reverse reads were respectively trimmed at 220 and 210 bp length in order to discard the low quality parts of the sequences. Lastly, chimera were removed. 11 992 816 reads remained out of 17 555 706. The lowest number of reads per sample was 1106, the highest was 132 274. All samples combined, a total of 65 169 Amplicon Sequence Variants (ASVs) was obtained. Those ASVs were compared to the Silva database (version 138.1) with the *assignTaxonomy* function in order to obtain the taxonomic identification of ASVs. The bootstrapping threshold was set to 100. Sequences identified as belonging to the genus *Pseudarcicella* were then cross-referenced with the NCBI database using the BLAST tool (https://blast.ncbi.nlm.nih.gov), and subsequently reassigned to the *Aquirufa* genus. This change in identification is explained in the section “Discussion.” Afterwards, eukaryotes, chloroplasts, mitochondria, and archaea were discarded. 9528 216 reads (79,4%) corresponding to 31 578 bacterial ASVs (i.e. 48,5%) remained after this step. To perform alpha diversity comparisons, a random rarefaction of the ASV abundances was conducted using the “rrarefy” function (Oksanen et al. 2019). Prior to rarefaction, any sample containing <10 000 reads was excluded from the analysis to preserve sufficient diversity information. This exclusion resulted in the loss of 14 samples out of 205 (7% of the samples), including four samples of the spatial campaign on the MR, three of the temporal campaign on the MR, and seven of the spatial campaign on the HW. 10 out of the 14 samples corresponded to LF samples. The rarefaction process was then carried out using the lowest number of reads among the 190 remaining samples, which was 10 019. The list of all ASVs and their abundance in those 190 samples with the taxonomy associated can be found in Table S3.

### Statistical analyses

Alpha diversity was assessed based on the Shannon index calculated using the Vegan package in R (Oksanen et al. 2019). Depending on data normality distribution, ANOVA or Kruskal–Wallis tests were applied to compare alpha diversity values between groups (SF spring versus LF spring, SF spring versus LF summer,…). To define whether alpha diversity values were significantly linearly correlated with distance from the river mouth, Pearson correlation coefficients were calculated. Spearman rank correlations were also carried out to determine potential correlations between Shannon indexes or the most abundant genera (top 20) and physico–chemical parameters.

The top 20 genera were determined separately for three distinct groups: spatial MR, spatial HW, and temporal campaign. In both spatial groups, the top 20 genera were calculated by aggregating data from the SM and LF, as well as from both spring and summer seasons. Similarly, for the temporal campaign, data from both fractions were aggregated to determine the top 20 genera.

For beta diversity calculations, no rarefaction was performed. Instead, data were processed according to Gloor et al. (2017). First, the function cmultRepl from the *zCompositions* package (v1.3.4) was used. This function enables to transform ASVs with zero count (which could cause errors after log-ratio transformation), into near-zero estimates (or probability of occurrence), therefore considering undersampling instead of absence. Afterwards, the *microbiome* package was used to perform a centered log-ratio transformation (clr) (Aitchinson 1986).

A PERMANOVA test (adonis2 function in R) was performed to identify, which variables explained beta diversity among the parameters measured (i.e. most abundant genera and physico–chemical parameters). Distance-based constrained analysis of redundancy (RDA) was then executed using the Vegan package to represent the differences in beta diversity between the different groups of samples (spatial, temporal, SF, LF,…). The physico–chemical parameters measured during both the spatial and temporal campaigns were represented on the RDA plot as vectors, with their length positively correlated to the *R*^2^ values of the PERMANOVA test.

## Results

### Patterns of alpha diversity in the HW and along the MR

**Figure 2.**
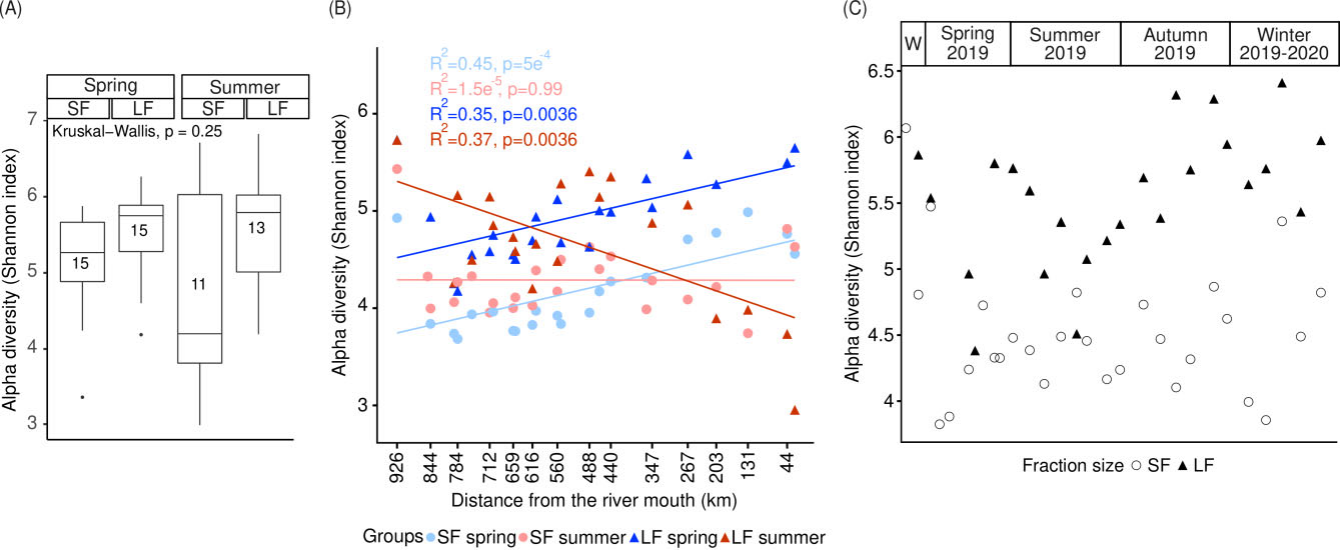
Alpha diversity at the ASV level—(A) in HW, (B) in the Meuse MR (spatial study), and (C) at Jambes (annual study), W = winter 2018-2019. Numbers in the boxplots of Fig. 2(A) represent the numbers of samples. *R*^2^ and *P*-values of Fig. 2(B) correspond to the results of the Pearson correlation tests. Not all sample kms are present on the *x*-axis of Fig. 2(B) to keep it readable. Km 926 corresponds to the river source.

In the HW, no difference in alpha diversity was observed between spring and summer nor between SF and LF bacterial communities (Kruskal–Wallis test, *P*-value = .25) (Fig. 2A).

Median Shannon index values were 5.3 for SF spring, 5.7 for LF spring, 4.2 for SF summer, and 5.8 for LF summer. Variations in alpha diversity was relatively consistent across the four groups, except for SF summer, which showed greater variation. The lower number of samples for the summer season was both due to low sequencing coverage (which led to discard those samples, see the section “Materials and methods”) and the impossibility to sample some streams because they were dry.

Along the main stretch of the river, alpha diversity of the LF was greater than that of the SF, except close to the mouth of the river during summer, where the opposite trend was observed (Fig. 2B). Of note, the river source (first sampling point on the MR axis also included in the HW) was characterized by a much higher alpha diversity (Shannon index from 4.9 to 5.7) than the following sampling site located around 69 km downstream.

**Figure 3.**
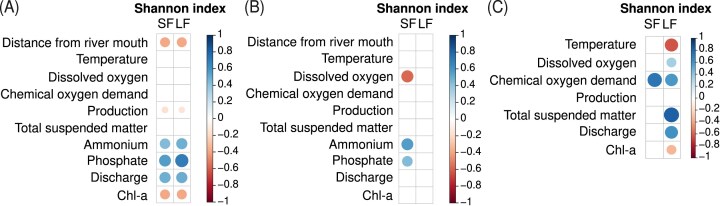
Spearman correlation matrices between physico–chemical parameters and the Shannon diversity index of the SF and LF fractions. (A) Spatial study in spring (without HW). (B) Spatial study in summer (without HW). (C) Temporal study. The scale and therefore the size of the dots corresponds to the value of the Spearman correlation coefficient (*ρ*). Only correlations with *P*-value < .05 are represented by a symbol.

During spring, alpha diversity of the LF and SF fractions increased significantly along the river course (respectively *R*^2^ = 0.35, *P*-value = .0036; *R*^2^ = 0.45, *P*-value = 5e^−4^) (Fig. 2B). This could be put in relation with the river discharge that was much higher during the spring campaign than the summer one and increased sharply downstream (Fig. S1A). This negative correlation between the Shannon index (for both fractions) and the distance from the river mouth or river discharge in spring was further confirmed by calculations of Spearman correlation coefficients (*ρ* = −0.49, *P*-value = .0042 for SF; *ρ* = 0.49, *P*-value = .0064 for LF) (Fig. 3A). Moreover, for both fractions, a positive correlation was also observed between the Shannon index, phosphate (*ρ* = 0.55, *P*-value = .0003 for SF; *ρ* = 0.69, *P*-value = .0001 for LF), and ammonium (*ρ* = 0.63, *P*-value = .046 for SF; *ρ* = 0.49, *P*-value = .034 for LF), and a negative one with Chl-a (*ρ* = −0.38, *P*-value = .009 for SF; *ρ* = −0.38 *P*-value = .015 for LF).

During summer, a significant decrease in alpha diversity occurred in the LF along the main axis (*R*^2^ = 0.37, *P*-value = .0036), with a major drop between km 267 and km 202 from the river mouth (Fig. 2B). On the contrary, no significant variation of alpha diversity could be observed for the SF along the main axis (*R*^2^ = 1.5e^−5^, *P*-value = .99) (Fig. 2B). Calculations of Spearman correlation coefficients revealed that the alpha diversity of the SF was significantly correlated to ammonium (*ρ* = 0.55, *P*-value = .0034) and phosphate (*ρ* = 0.43, *P*-value = .037), and negatively to DO concentration (*ρ* = −0.57, *P*-value = .012) (Fig. 3B). No correlation of alpha diversity was observed with river discharge.

### Evolution of alpha diversity over 1 year at one sampling site

Generally speaking, the Shannon index of bacterial communities at the station sampled every second week for 1 year (Jambes) was again higher for the LF than the SF (Fig. 2C). Both fractions were characterized by notable variations of alpha diversity between successive sampling dates. Nevertheless, a decreasing pattern in alpha diversity of the LF could be highlighted during summer, followed by an increase at the end of the summer, in autumn and in winter (Fig. 2C), to values above those observed in the two spatial campaigns on the MR axis (Fig. 2B). No clear pattern could be highlighted for the SF. Interestingly, the high values of alpha diversity of the LF fraction in autumn and winter matched those of river discharge at the same seasons (Fig. S1B). The link between both variables was confirmed by the positive Spearman correlation between the Shannon index of the LF and river discharge (*ρ* = 0.6, *P*-value = .002) (Fig. 3C). In addition, the Shannon index of the LF was positively correlated to the concentration of TSM (*ρ* = 0.81, *P*-value = .00001), COD (*R*^2^ = 0.57, *P*-value = .0487), and DO (*ρ* = 0.34, *P*-value = .032); differently, it was negatively correlated to temperature (*ρ* = −0.62, *P*-value = .0026) and Chl-a (*ρ* = −0.33, *P*-value = .0118) (Fig. 3C). Concerning the SF, the only significant correlation was that of the Shannon index with the COD (*ρ* = 0.73, *P*-value = .008).

### Evolution of the 20 most abundant genera across the watershed and along the year

**Figure 4.**
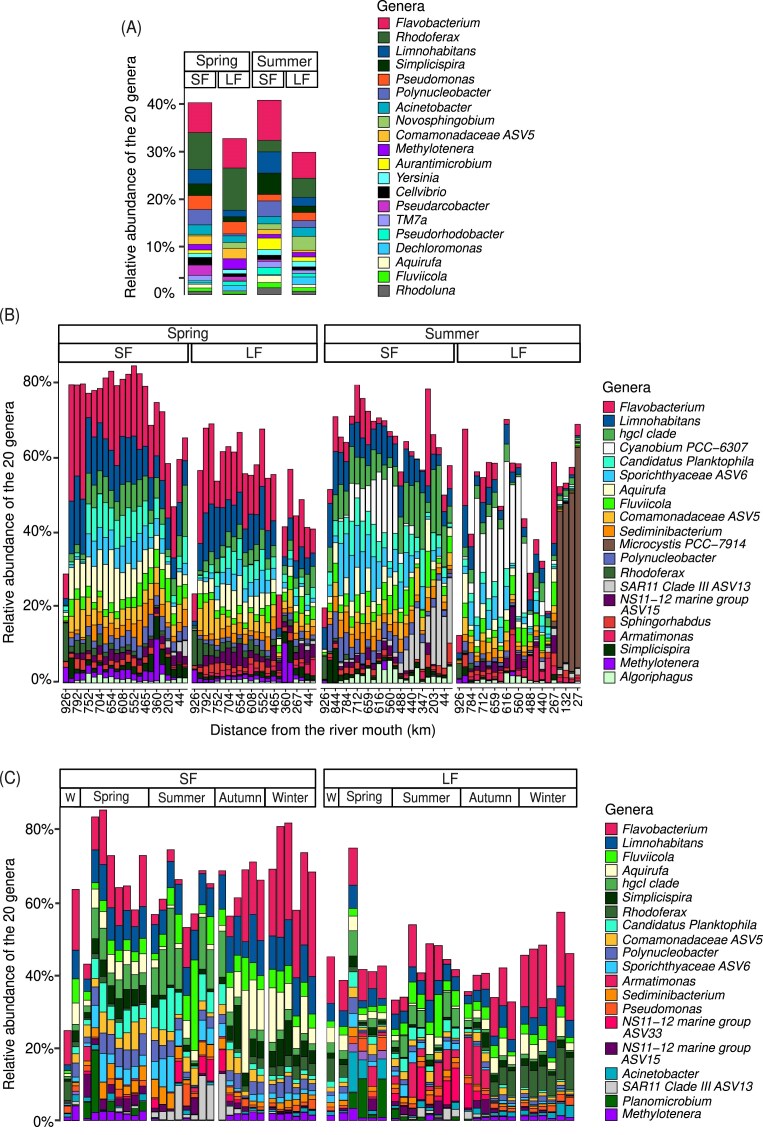
Relative abundance of the top 20 genera (A) in the HW, (B) in the main fluvial axis, and (C) in the temporal study at Jambes. W = winter. Not all sample kms are present on the *x*-axis of Fig. 4(B) to keep it readable. Km 926 corresponds to the river source.

**Figure 5.**
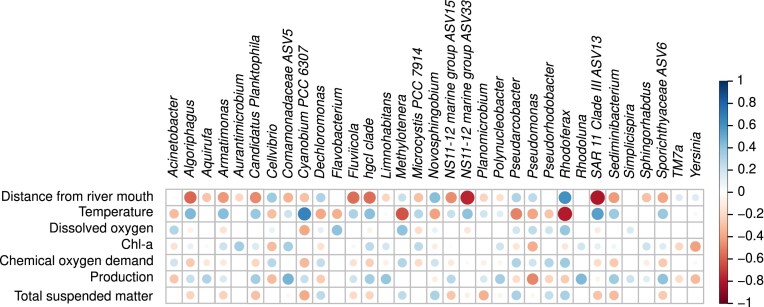
Spearman correlation matrix between (a)biotic parameters and the 20 most abundant genera (that were calculated separately for HW–MR–Temporal and further aggregated). The scale and therefore the dot sizes correspond to the value of the Spearman correlation coefficient (*ρ*). Only correlations with a *P*-value <.05 are represented by a symbol. Taxa for which the PERMANOVA *P*-value was not significant (Table 2) have been removed.

We then identified separately the 20 most abundant genera in the HW (Fig. 4A), along the Meuse main axis (MR) during spring and summer (Fig. 4B), and over 1 year at Jambes (Fig. 4C). All in all, this represented 33 different genera when the different studies were aggregated (Fig. 5).

In the HW, the top 20 most abundant genera accounted for 30%–40% of all ASVs (Fig. 4A), whereas they represented a more variable percentage of all ASVs (20%–80%) in both the MR and the temporal study (Fig. 4B and C).

Nine genera were unique to the top 20 of HW (i.e. not found in the top 20 of the MR or of the temporal study). Those were, in decreasing order of abundance, *Novosphingobium, Aurantimicrobium, Yersinia, Cellvibrio, Dechloromonas, Pseudarcobacter, TM7a, Pseudorhodobacter*, and *Rhodoluna. Novosphingobium, Dechloromonas, Pseudarcobacter*, and *Pseudorhodobacter* were negatively correlated with temperature and positively with TSM, whereas *Cellvibrio* was negatively correlated with temperature but not with TSM (Fig. 5).

Nine top 20 genera were shared by the different studies (spatial HW, spatial MR, and temporal studies), representing almost half of the top 20 of each study. Among them, two genera, *Flavobacterium* and *Limnohabitans* were especially abundant across in all studies. The other ones were Comamonadaceae ASV5, *Fluviicola, Methylotenera, Polynucleobacter, Aquirufa, Rhodoferax*, and *Simplicispira. Rhodoferax* was more abundant in the HW, in both fractions and at both seasons, with a predominance in spring. A peak of this genus could also be observed during late autumn and winter in the LF (and to a lower extent in the SF) of the temporal study. Finally, it was easily detected in both fractions of the MR until km 203 (from the river mouth) in spring. *Rhodoferax* was negatively correlated with temperature and positively with TSM (Fig. 5). *Flavobacterium* was the second most abundant genus in the HW in spring and first one in summer (Fig. 4A). Moreover, it was very abundant in the MR in spring (Fig. 4B), and during autumn and winter in the temporal study (Fig. 4C). In summer, its relative abundance was greatly reduced in the MR and at Jambes. Coherently, it was negatively correlated to temperature (Fig. 5). This taxon showed no preference for either of the fractions. *Limnohabitans* was more abundant in the SF than the LF in the HW during both seasons. Similarly, it was very abundant in the SF of the MR at both seasons, as well as in summer in the LF. It remained stable throughout the temporal study for both fractions. Furthermore, its relative abundance decreased along the MR whatever the season or fraction (Fig. 4B). This change in relative abundance with distance was confirmed by a Spearman correlation (Fig. S2). Concerning *Aquirufa*, its was little detected in the HW. In the MR and at Jambes, it was more abundant in the SF than LF, and consistently detected in spring, autumn, and winter while its presence in summer was sporadic (Fig. 4B and C). *Fluviicola* and *Polynucleobacter* were present at all stations along the MR and throughout the year at Jambes, with no clear variation in relative abundance according to fraction or season for the first one, the second one being more abundant in the SF fraction. Comamonadaceae ASV5 was consistently detected in spring in the HW, MR, and temporal study, and in lower abundance in summer and autumn. Generally speaking, it was more abundant in the SF than the LF. *Methylotenera* was detected in spring in the spatial campaigns (HW and MR), and in low abundance in autumn, winter, and spring at Jambes. It was largely absent from the water masses in summer and exhibited a strong negative correlation with temperature (Fig. 5). Finally, *Simplicispira* was recovered in greater abundance in the SF than the LF whatever the study. It was less abundant in summer than in the other seasons.

In addition, seven top 20 genera were shared by the MR and the temporal study but not detected in the 20 dominant taxa in HW, which means that the water masses along the MR and at Jambes shared a majority (16) of their top 20 genera. Those seven genera were *Armatimonas, Candidatus Planktophila, hgcI clade*, NS11-12 ASV15, SAR11 Clade III ASV13, Sediminibacterium, and Sporichtyaceae ASV6. All were positively correlated with temperature and negatively with TSM (Fig. 5). In agreement with those results, *Candidatus Planktophila, hgcI clade*, and Sporichtyaceae ASV6 shared similar spatio–temporal patterns, i.e. a greater relative abundance in summer. In addition, they were more abundant in the SF over the LF. Those trends were also verified in the temporal study, in which those three taxa were almost absent in winter. *Armatimonas* was more abundant in the LF fraction in all sampling campaigns, especially in summer and autumn. SAR11 Clade III ASV13 was largely represented in the SF of the MR (where a steady increase in abundance was observed downstream, from km 465 to km 27 of the river mouth), and of the temporal study in summer.

Finally, two taxa were greatly present in the top 20 of the MR in summer and not in the other groups: *Cyanobium PCC-6307* and *Microcystis PCC-7914* (Fig. 4B). Both taxa were positively correlated to temperature (Fig. 5). A positive correlation was also observed between Cyanobium *PCC-6307* and TSM. *Cyanobium PCC-6307* was the most dominant genus in the upper part of the MR (from km 752 to km 552 from the river mouth) in both fractions (10%–37%), and *Microcystis PCC-7914* dominated downstream in the LF (from km 203 to km 27 of the river mouth). At the last four stations, this sole genus represented 50%–65% of all ASVs in the LF.

### Driving parameters of beta diversity patterns

Table 1 presents the ranges of abiotic and biotic parameters measured for the three campaigns: HW during spring and summer, MR during spring and summer, and the temporal study at Jambes.

**Table 1.** Ranges (min–max) of abiotic and biotic parameters measured for the three campaigns: HW during spring and summer, MR during spring and summer, and the temporal study at Jambes.

Parameters	HW		MR		Temporal
	Spring	Summer	Spring	Summer	
**Temp (°C)**	6.5–16.2	13–19.4	10–16.4	15.7–22.7	6.4–23.3
**DO (ppm)**	2.83–11.41	1.8–9.74	7.84–13.39	3.71–8.27	7.1–13.44
**COD (mg O_2_/l)**	4–92.4	2.04–76.7	0.3–85.1	0.1–66.8	5.8–38.11
**Production (µg C/l/h)**	0.002–0.114	0.002–0.627	0.009–0.184	0.013–0.214	0.01–0.1
**TSM (mg/l)**	1.4–38.7	2.3–58.3	2.6–18.3	2.3–16.04	1.4–82.4
**Chl-a (µg/l)**	0.21–17.5	0.17–13.89	0.4–18.3	0.465–29.17	0.2–59.6
**Ammonium (mg/l)**	0.016–6.927	0.008–0.102	0.022–0.442	0.027–0.315	NA
**Phosphate (mg/l)**	0.004–2.223	0.03–1.77	0.01–0.4	0.02–0.91	NA
**Discharge (m^3^/s)**	NA	NA	0.01–188.33	0.01–43.695	22.1–786.38

Temp = temperature, DO = dissolved oxygen, COD = chemical oxygen demand, TSM = total suspended matter, and Chl-a = chlorophyll *a*.

A PERMANOVA test was done to identify which (a)biotic parameters explained the dissimilarity between bacterial communities, all samples pooled. The test was significant for all physico–chemical parameters (*P*-value < .05), and the best explanatory ones (*R*^2^ > 0.05) were season, temperature, and distance from the river mouth (Table 2). Moreover, among the top 20 most abundant taxa calculated separately for all three datasets (HW, main fluvial axis, and temporal study) and aggregated, *Flavobacterium* emerged as the most influential taxon to discriminate the communities, followed by *Rhodoferax* and *Sediminibacterium*.

**Table 2.** PERMANOVA analysis to identify the driving parameters of beta-diversity among the physico–chemical parameters and among the top 20 most abundant genera (that were calculated separately for HW–MR–Temporal and further aggregated).

Parameters	*R* ^2^	*F*	*P*-values	Parameters	*R* ^2^	*F*	*P*-values
*Flavobacterium*	0.3012	5.031	.001	*NS11-12 marine group ASV3*	0.02 503	4.158	.001
**Season**	0.12 422	4.255	.001	*SAR 11 Clade III ASV13*	0.0243	4.034	.001
**Temperature**	0.07 308	14.585	.001	*Armatimonas*	0.02 383	3.553	.001
*Rhodoferax*	0.05 881	10.122	.001	*Yersinia*	0.02 354	3.906	.001
*Sediminibacterium*	0.053	9.066	.001	*Sphingorhabdus*	0.02 158	3.573	.001
**Distance from river mouth**	0.05 239	10.228	.001	*Pseudarcobacter*	0.02 042	3.377	.001
*Candidatus planktophila*	0.05 105	8.715	.001	*Microcystis PCC 7914*	0.01 933	3.193	.002
*hgcI clade*	0.04 804	8.175	.001	*Planomicrobium*	0.01 926	3.181	.001
*Fluviicola*	0.04 639	7.881	.001	*Novosphingobium*	0.01 853	3.058	.001
**Fraction size**	0.04 496	8.709	.001	*Comamonadaceae ASV5*	0.01 556	2.560	.001
*Limnohabitans*	0.04 372	7.407	.001	*Acinetobacter*	0.01 534	2.523	.002
*Sporichthyaceae ASV6*	0.04 231	7.158	.001	*Rhodoluna*	0.01 507	2.479	.004
*NS11-12 marine group ASV15*	0.04 056	6.848	.001	*TM7a*	0.01 416	2.326	.003
*Cellvibrio*	0.03 779	6.362	.001	*Methylotenera*	0.01 333	2.189	.009
*Dechloromonas*	0.03 751	6.313	.001	**COD**	0.01 202	2.044	.001
*Algoriphagus*	0.03 728	6.273	.001	*Simplicispira*	0.01 117	1.829	.018
**DO**	0.03 673	7.053	.001	*Polynucleobacter*	0.01 116	1.828	.057
*Aquirufa*	0.03 652	6.140	.001	**Chl-a**	0.01 105	1.810	.021
*Pseudomonas*	0.03 456	5.798	.001	**Production**	0.00 988	1.617	.037
*Cyanobium PCC 6307*	0.02 906	4.849	.001	*Aurantimicrobium*	0.00 983	1.608	.09
**TSM**	0.02 536	4.214	.001	*Pseudorhodobacter*	0.00 943	1.543	.147

Parameters are ranked according to decreasing *R*^2^ values. Genera are in italic and (a)biotic parameters in bold. Ammonium and phosphate are not present in the table as not measured during temporal campaign, and river discharge neither, as not measured in HW.

**Figure 6.**
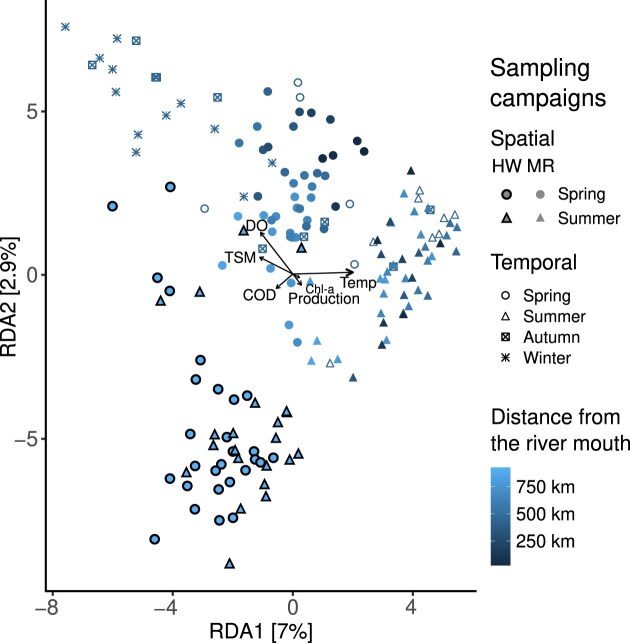
RDA representing all samples (HW, spatial studies on the MR, and temporal study). HW = headwaters; MR = main river. (A)biotic parameters measured are presented as vectors. Temp = temperature; TSM = total suspended matter; DO = dissolved oxygen; COD = chemical oxygen demand; and Chl-a = Chlorophyll *a*.

On the redundancy analysis plot (Fig. 6), a progressive distinction of samples according to distance and season was visible for the spatial surveys, with the HW samples being clearly separated from the others. Regarding the temporal survey, most samples of the autumn and winter seasons formed a cluster separated from the spatial study, revealing a different BCC during those seasons, while the summer and spring samples were grouped with the spatial samples of the same seasons. The temperature vector pointed toward the summer season downstream (and concomitantly to the summer samples of the temporal campaign), whereas the DO and TSM vectors pointed toward the autumn and winter samples of the temporal campaign. The latter vector also pointed toward some HW samples. The bacterial production and Chl-a vectors pointed toward midstream in summer, and the COD vector pointed toward the upstream samples of the spatial campaign. Despite being significant according to the PERMANOVA test (Table 2), the factor “fraction size” did not differentiate the samples as strongly as season, temperature, and distance (Fig. S3).

### Identification of bacterial taxa correlated with physico–chemical parameters of water quality

In order to identify taxa among the top 20 most abundant ones that were correlated with physico–chemical parameters indicative of water quality, i.e. DO, ammonium, and phosphate concentrations, we further analyzed the Spearman correlation matrix of Fig. 5. Ten genera were identified as positively correlated with DO. The most significant correlations were observed for *Flavobacterium* and *Rhodoferax* (two discriminating taxa of beta diversity, Table 2) and *Methylotenera*. In addition, a strong negative correlation was observed between DO and *Cyanobium PCC 6307* (Fig. 5). Another Spearman correlation matrix was calculated exclusively on the main fluvial axis spatial study (Fig. S2), during which the most abundant taxa exhibited great variations (Fig. 4B) and for which nutrient concentrations were available. Here again, the three abovementioned taxa (*Flavobacterium, Rhodoferax*, and *Methylotenera)* showed a strong positive correlation with DO, and the negative correlation between *Cyanobium PCC 6307* and DO was confirmed. In addition, *Flavobacterium* and *Rhodoferax* were negatively correlated with phosphate concentration (Fig. S2). Plus, the relative abundance of *Rhodoferax* was positively correlated to COD, a proxy of the amount of organic matter in the water (Fig. 5 and Fig. S2). Other genera were correlated with DO and nutrients in the spatio–seasonal campaigns of the Meuse River axis. On one side, *Limnohabitans, Aquirufa*, Comamonodaceae *ASV5*, and *Sphingorhabdus* were positively correlated with DO and negatively correlated with phosphate (Fig. S2). On the other side, SAR11 Clade III ASV13 and *Microcystis PCC 7914* were positively correlated with phosphate concentration.

## Discussion

### The LF holds greater diversity than the SF

In this survey of the Meuse watershed, both spatially (at two seasons) and temporally (at one sampling station mid-stream, Jambes), alpha diversity was significantly greater in the LF than in the SF. Such a trend was observed in the HW (although not significant), in the waters of the main fluvial axis (with the exception of the summer samples close to the mouth, dominated by *Cyanobacteriota*), and at Jambes throughout the year. The difference in bacterial diversity according to fraction size is in line with previous studies, which addressed this topic (Crump et al. 1998, Mohit et al. 2014, Rieck et al. 2015, Payne et al. 2017, 2020, Gweon et al. 2021). It is generally explained by the nutrient-rich and varied microenvironments associated with particles, which tend to harbor more diverse microbial communities than the free-living communities (Wang et al. 2012). On the other hand, the dominance of two *Cyanobacteriota* genera in the LF of some samples is likely due to their ability to form microcolonies, with an average size of 40 µm for *Cyanobium* (Jezberová and Komárková 2007) and >100 µm for *Microcystis* (Xiao et al. 2018).

Despite significant differences in alpha diversity between the SF and the LF, numerous taxa were shared between the two fractions (i.e. *Flavobacterium, Limnohabitans, Aquirufa*, Sporichthyaceae ASV6, and Comamonadaceae ASV5). Indeed, many bacterial taxa can alternate between free-living and particle-associated lifestyles (Grossart 2010). However, notable differences in BCC between SF and LF can also be highlighted in this study. Indeed, some taxa were far more present in the SF (i.e. *hgcI clade, Polynucleobacter*, and SAR11 clade III ASV13), whereas other taxa were in the LF (i.e. *Armatimonas* and *Microcystis PCC-7914*). Similar results were reported by Jackson et al. (2014), which observed a prevalence of the *Cyanobium* clade in the larger fraction of water masses in the Mississippi watershed in summer, while the SAR11 clade was predominantly found among bacteria of smaller fraction sizes. In addition, consistent with our findings, Savio et al. (2015) observed a dominance of the SAR11 clade and *hgcI* clade in the SF in the Danube River in summer. The ecology of several of these taxa is discussed further below.

### Bacterial alpha diversity changes from HW to the mouth of the Meuse River

Unlike waters of the main axis, the HW did not show significant change in alpha diversity between season or fraction, revealing a stable diversity of the water masses. Our results are in contrast with another seasonal study on HW, where a higher diversity was observed during spring compared to summer, which was explained by the higher influence of allochthonous inputs during spring (Laperriere et al. 2020).

In addition, the Shannon index of HW (around 5.5) was higher than that of the waters of the main axis (mostly between 4 and 5). Of note, the sharp decrease in alpha diversity that was observed between the HW (including the Meuse source, km 926 of the river mouth) and the second sampling point along the Meuse axis located 69 km downstream indicates that this stretch of the river deserves further exploration in the future, with sampling at intermediate locations. Nevertheless, the greater alpha diversity of HW compared to locations further downstream has been observed in the Danube as well (Savio et al. 2015). It was explained by the mass effect being a bigger driver of diversity upstream than species sorting. Moreover, groundwater has been reported to hold a greater bacterial diversity than river water (Retter et al. 2023, Ji et al. 2022). This difference is explained by a more neutral pH (Fierer et al. 2007) and a more stable temperature of groundwater (Pinto and Nano 2015). Finally, Retter et al. (2023) highlighted that the greater productivity (based on cellular ATP and cell count) in river compared to groundwater results in a lower diversity, which is consistent with our findings.

In the spring campaign, the increase in alpha diversity along the main axis could be explained by higher precipitations than during that of summer. As a consequence of precipitations, a steep, progressive increase of discharge was observed along the main axis. The positive correlation between alpha diversity and discharge could be explained by the fact that the dispersion effect overrode the species sorting effect during rainfall events. This hypothesis was put forward in the temporal study of a Canadian river subject to seasonal ice cover by Cruaud et al. (2020) and was also supported by the work of Caillon et al. (2021) on the effect of flood events on the BCC of streams.

Conversely, the decrease in diversity along the main axis observed in summer (for LF) was consistent with another study carried out at this season in other rivers in the Northern hemisphere (Ruiz-González et al. 2015). In our study, the summer campaign was characterized by a much lower flow than the spring one, and it is likely that species sorting overrode mass effect.

### Bacterial alpha diversity varies substantially over 2-week intervals at the same sampling station

Significant fluctuations in alpha diversity were observed within a 2-week period throughout the year. Similar observations were made in an annual study conducted at a single sampling site on the Mississippi River by Payne et al. (2020). The authors explained those short-term fluctuations, especially noticeable in the summer, by sudden and unpredictable disturbances happening briefly (such as variations in local currents).

Seasonal variations were noticed in our study as well: alpha diversity of the LF increased with river discharge (especially in winter). This rise in alpha diversity was most likely linked to a rise in the concentration of suspended particulate matter carried in the water during high water events, providing additional microhabitats for the bacteria (Crump et al. 1999, Ortega-Retuerta et al. 2013). Indeed, a high correlation coefficient was recorded between alpha diversity of the LF and TSM in our study. The decrease in alpha diversity during summer was expected, as species sorting is known to be positively correlated with temperature (Wang et al. 2019).

### The dominant genera unique to HW are not typical freshwater taxa

As mentioned earlier, Novosphingobium, Aurantimicrobium, Yersinia, Cellvibrio, Pseudarcobacter, TM7a, Pseudorhodobacter, Dechloromonas, and Rhodoluna were detected in the top 20 most abundant genera of HW but not of the MR axis. Novosphingobium is a ubiquitous, metabolically versatile taxon that has been found in a large variety of habitats, where it decomposes organic compounds (including pollutants): the rhizosphere, contaminated bulk soils, seawater, and freshwater (Lee et al. 2014, Sheu et al. 2016, Kumar et al. 2017). The type strains of Aurantimicrobium have been isolated from various habitats such as freshwater (Nakai et al. 2015), a river receiving swine wastewater (Sun et al. 2024) and fish gut microbiota (Chen et al. 2024). Yersinia, has been detected in various environments, such as human feces, animal feces and intestines, freshwater, and food (Sulakvelidze 2000, Fukushima et al. 1988). *Cellvibrio* is a genus associated to sediment, soil, and rhizosphere environments (Blackall et al. 1985, Mergaert et al. 2003, Zhang et al. 2020, Lau and Furusawa 2024), with exceptional capabilities to degrade plant biomass (Xie et al. 2017, Lau and Furusawa 2024). While it has been observed in localized HW in the Southeastern USA (Teachey et al. 2019) and in natural springs in Taiwan (Chen et al. 2017), its absence from studies performed on a broader scale like that of Laperrière et al. (2020) in Northeastern USA streams suggests that its distribution may be site-specific and heavily influenced by local environmental factors. The presence of Dechloromonas is often associated with anoxic, organic-rich environments such as Wastewater Treatment Plants (WWTPs) (Hu et al. 2012, Saunders et al. 2016). *Pseudarcobacter* has been detected in a variety of aquatic environments such as seawater, marine invertebrates, but also sewage and WWTPs (Basiry et al. 2024). Similarly, *Pseudorhodobacter* has been recovered from marine sediment, seawater, marine invertebrates, but also wastewater (Bian et al. 2024) and sludge (Calderon-Franco et al. 2022). TM7a has been found in soils, the human gut, and riverine environments (Jin et al. 2024). Lastly, Rhodoluna is an aquatic genus that has been reported across the Danube River (Fontaine et al. 2023) but was only identified here as part of the top 20 genera of HW and not of MR.

In conclusion, most genera exclusive to the HW of the Meuse watershed are associated to soil and/or aquatic environments predominantly rich in organic matter. This observation aligns with the greater values of COD recorded in the HW samples compared to those of the MR. In addition, the association of several of those taxa with wastewater/sludge suggest a potential contamination of HW sampling sites by wild animal feces, cattle, or maybe human wastewater. However, those results should be interpreted with caution, as many of the abovementioned bacterial genera include multiple species with different ecological niches.

### Several dominant taxa detected along the MR axis and in the temporal study were identified as potential bioindicators of water quality

Three broad-ranging parameters were selected to assess water quality within the Meuse watershed: DO, ammonium, and phosphate concentrations. In the case of the Meuse River, Chl-a could not serve as a proxy of eutrophication, and thus as an indicator of river quality, due to its reduction by the activity of filter-feeding invasive species (discussed in detail further).

Two dominant taxa in the temporal and spatial studies (MR and HW) were identified as potential indicators of water quality in the Meuse watershed, as was the case in studies on other watersheds: *Flavobacterium* and *Aquirufa*. Regarding *Flavobacterium*, the prevalence of this primarily aerobic chemoorganotroph genus can be attributed to its capacity for degrading a range of biopolymers like cellulose, chitin, and pectin (Kirchman 2002). In the Meuse River, *Flavobacterium* can be considered as an indicator of good water quality, due to its positive correlation with DO and negative correlation with phosphate. The same status was inferred by Fontaine et al. (2023) in the Danube River. Its almost complete disappearance from the top 20 genera in the main axis of the Meuse River in summer is in line with results reported in the Mississippi River (Payne et al. 2020). One possible explanation can be found in the negative correlation of *Flavobacterium* with temperature in the Meuse fluvial axis.

As for *Aquirufa*, it has recently been isolated from freshwater environments closely linked to terrestrial ecosystems. It has the ability to degrade pectin, a polymer found in the cell walls of terrestrial plants (Pitt et al. 2019, 2022, Sheu et al. 2020). Moreover, its rhodopsin system allows it to perform photoheterotrophy (Pitt et al. 2022), enabling survival in nutrient-poor environments (Chiriac et al. 2023). It is suspected to be a prevalent freshwater taxon, as the 16S rRNA gene of isolates match that of uncultured clones found in various studies on rivers (Crump and Hobbie 2005), lakes (Burkert et al. 2003), and freshwater sediments (Tamaki et al. 2009). *Aquirufa* is closely related to *Pseudarcicella*, a genus initially isolated from leech skin (Kämpfer et al. 2012) and commonly identified in various riverine environments (Sun et al. 2018, Yang et al. 2019, Cruaud et al. 2020). It should be noted that *Aquirufa* can be mistaken for *Pseudarcicella* during routine identification with certain databases (Hahn M, personal communication), highlighting the importance of careful examination of ASV sequences. *Aquirufa* was considered an indicator of good water quality in the Meuse due to its positive correlation with DO and negative correlation with phosphate. This aligns with previous findings, which reported a strong negative correlation between *Aquirufa* abundance and total algae levels in a lake reoligotrophication assessment (Farkas et al. 2022).

In addition to those three taxa, it is noteworthy to highlight the presence of the genus *Rhodoferax* in the top 20 most abundant genera of the Meuse watershed, especially the HW and the temporal campaign. *Rhodoferax spp*. are purple nonsulfur, mostly facultative anaerobic bacteria (Kaden et al. 2014). This genus was defined as a “typical freshwater taxon” (Okafor 2011), which reduces iron. It represented ∼5% to ∼10% of all ASVs at the river source, which aligns with multiple previous studies reporting *Rhodoferax* in aquifers (Zhuang et al. 2011, Abiriga et al. 2021, Kasanke et al. 2021). Species sorting would then make it progressively decrease in the next kilometers, which was observed from the second sampling point along the river axis, especially in summer. Therefore, it can be considered a tracer of groundwater. *Rhodoferax* relative abundance was negatively correlated to temperature and phosphate concentration, two parameters increasing downstream. Its greater presence during late autumn and winter is coherent with a recent study on Chinese urban rivers (Wang et al. 2023) and with the identification of this genus in cold environments such as Arctic lakes (Van Trappen et al. 2002), beneath an Arctic glacier (Cheng and Foght 2007), and in the permafrost (Steven et al. 2008).

Furthermore, two taxa were observed at specific locations and seasons, with a status of indicator of poor water quality that is backed up by the literature: *Cyanobium PCC-6307* and *Microcystis PCC-7914*. Those *Cyanobacteriota* genera were detected in great abundance in the LF fraction of downstream locations along the fluvial axis in summer, concomitantly with the highest values of Chl-a within MR (max 30 mg/l). However, no statistical correlation between those *Cyanobacteriota* relative abundances and Chl-a concentrations could be established. Phytoplankton blooms (∼120 mg of Chl-a/l) used to occur in the upstream section of the Meuse (until km 400 from the river mouth), where the discharge is still moderate (Descy et al. 1987). Indeed, phytoplankton production depends on the balance between growth rate and dilution rate. Downstream, the phytoplankton biomass would decrease due to dilution by tributaries, protozoan grazing, and cell mortality (Descy and Gosselain 1994). However, this pattern is no longer valid, as blooms have drastically diminished in the Meuse River for the last 15 years due to the invasion of filter-feeding molluscs such as *Dreissena polymorpha* (zebra mussel), *Dreissena polymorphys* (Marescaux et al. 2015), and *Corbicula* spp. (Pigneur et al. 2014). The greater abundance of *Cyanobium* midstream could be negatively and positively correlated with DO and temperature, respectively. In accordance with this, *Cyanobium* has been described as frequently found in warm waters (Stanier et al. 1971). Moreover, the presence of *Cyanobium PCC-6307* has been reported in a variety of aquatic environments, such as lakes and reservoirs, rivers (Eraqi et al. 2021, Blais et al. 2022), coastal areas (Adyasari et al. 2020), seas (Kolda et al. 2020), or even WWTP effluents (Millar et al. 2022). *Microcystis PCC-7914* has been reported in a smaller range of habitats, i.e. lakes (Wu et al. 2021, Li et al. 2023) and rivers (Millar et al. 2022). Its peak close to the river mouth can be explained by its positive correlation with phosphate concentration (increasing downstream), which has already been highlighted in previous studies (Davis et al. 2009, Harke and Gobler 2013). Both *Cyanobium PCC-6307* and *Microcystis PCC-7914* are known to potentially release cyanotoxins (Millar et al. 2022); therefore their presence poses a health risk for the fauna and potentially humans and should be the object of further investigation.

Other taxa were identified as indicators in this study (i.e. *Limnohabitans, Methylotenera*, NS11-12 marine group, and SAR11 clade III) but their indicator status contradicted findings from previous studies. Detecting *Limnohabitans* among the dominant genera was unsurprising. Indeed, *Limnohabitans* has been characterized as a genetically diverse taxon with wide ecological distribution (Jezbera et al. 2013). Its significant contribution to freshwater bacterioplankton communities stems from its rapid substrate uptake and growth, utilization of algal-derived substrates, and susceptibility to high mortality rates from bacterivory (Kasalický et al. 2013). The extensive study on the Danube River of Fontaine et al. (2023) defined it as an indicator of eutrophic conditions, while here, the opposite status was suggested, due to its positive correlation with DO and negative correlation with phosphate. The difference might be due to the criterion used to identify bacterial indicators: Fontaine et al. (2023) looked for correlations of taxa abundance with Chl-a concentration (a proxy for eutrophication) whereas we proceeded in the same way using nutrient and DO concentrations. *Methylotenera* and NS11-12 marine group were other indicators of good quality in the Meuse watershed (positively correlated with DO and negatively with phosphate). However, previous studies have reported the presence of *Methylotenera* in numbers in rivers affected by agricultural activities (Huang et al. 2018), and the NS11-12 marine group was associated with metal contamination (Pb and Cu) in a coastal area (Coclet et al. 2019) and with dissolved organic carbon originated from algal blooms or from external inputs in lakes and rivers (Farkas et al. 2020).

The fourth taxon, SAR 11 clade III is typically associated with marine habitats but has also been detected in freshwater environments (Tsementzi et al. 2019). Its summer peak in the Meuse is in agreement with the results of several studies in oceans (Carlson et al. 2009, Eiler et al. 2009) and in lakes (Salcher et al. 2011, Heinrich et al. 2013). It might be linked to the presence of proteorhodopsin in these bacteria (Atamna-Ismaeel et al. 2008). Moreover, it presented a positive correlation with phosphate concentration, reflecting its potential as bioindicator of poor freshwater quality. This correlation is opposite (Salcher et al. 2011) or consistent (Heinrich et al. 2013) with what was observed in lacustrine environments.

Finally, regarding the other abundant genera across the Meuse watershed, we could not identify any as bioindicator. Some taxa (*Armatimonas, Candidatus Planktophila, hgcI clade*, and Sporichthyaceae ASV6) were not identified as bioindicators in the literature either, whereas others had been classified as indicators of good river quality, i.e. *Fluviicola* (Ji et al. 2018) and *Sediminibacterium* (Song et al. 2017), or of poor river quality, i.e. *Polynucleobacter* (Pandey et al. 2014) and *Simplicispira* (Vignale et al. 2023).

### Differences in BCC between spatial and temporal campaigns are mostly explained by season, distance, and temperature

Concerning the impact of season on beta diversity, it was expected to be significant. Indeed, the distinction between winter and summer samples, with spring and autumn intermediary, aligns with other studies (Crump and Hobbie 2005, 2009, Doherty et al. 2017, Payne et al. 2020). The second most influential physico–chemical parameter driving beta diversity was temperature. This has been shown to differentiate BCC in different studies of fluvial axes (Cruaud et al. 2020, Payne et al. 2020). The third driving parameter, distance from the river mouth, has been demonstrated to significantly influence BCC in various studies on temperate rivers undertaken during spring (Crump and Hobbie 2005, Jordaan and Bezuidenhout 2013, Read et al. 2015, Savio et al. 2015, Zhao et al. 2021). The same conclusion was drawn in a study on the Koshi River flowing through regions with cold to tropical climates (Paudel Adhikari et al. 2019). However, a recent investigation on the Nile River (Eraqi et al. 2021) revealed that distance did not influence beta diversity, neither during summer nor winter, presenting a notable deviation from previous findings. Finally, in our study, the impact of fraction size on beta diversity was lower than the driving parameters mentioned earlier, even if it was still significant. This result contrasts with several studies of riverine bacteria, which have shown a clear separation of samples according to the fraction size (Savio et al. 2015, Henson et al. 2018).

## Conclusion

This work was the first to address the BCC of the Meuse River watershed. Furthermore, its originality was to combine a spatio–seasonal survey with a high frequency annual survey. The taxa identified in the HW and the main Meuse River, at different time scales, were consistent with those found in other freshwater environments. Similarly, the main environmental parameters explaining the dissimilarity of BCC between sampling locations have been reported in other surveys of lotic bacterial communities. Yet, the riverine BCC in the Meuse watershed and its spatio–temporal variations were unique, further illustrating the absence of a single pattern of bacterial diversity in rivers worldwide. A notable distinction in our study was the relatively minor influence of fraction size on BCC variations compared to the more significant roles of season, temperature, and distance from the river mouth. This contrasts with other studies, which have placed greater emphasis on fraction size.

Moreover, some bacterial taxa were significantly correlated with physico–chemical parameters, highlighting their potential as indicators of good water quality in the Meuse River, i.e. *Flavobacterium, Limnohabitans, Aquirufa, Methylotenera, Rhodoferax*, and NS 11–12 marine group. Conversely, indicators of poor river quality could be identified as well, i.e. *Cyanobium PCC-6307, Microcystis PCC-7914* (particularly abundant in the summer campaign in the Meuse), and SAR 11 clade III. It is important to mention however, that the identification of those “bioindicator” genera was constrained by the limited number of physico–chemical parameters measured in this study. Moreover, ammonium and phosphate, were only measured in the spatial study on the MR axis due to technical limitations. To increase the discriminating power of such analyses, measurements of those parameters should be applied to any future study, and as well as other parameters, such as dissolved organic carbon and nitrate.

Additional spatial studies on this watershed during autumn and winter would be valuable to confirm the pronounced differences of BCC observed during the temporal campaign at those seasons. Further on, a multiyear analysis would provide a clearer understanding of the spatio–seasonal patterns in the Meuse watershed and potentially reveal the impact of climate change on riverine BCC. In that respect, performing analyses based on RNA sequencing of the 16S rRNA would provide an additional standpoint on the Meuse BCC, by allowing to identify the active fraction of the bacterial community inhabiting the water column. Lastly, metagenomic analyses would allow to characterize the key functions performed by the river microbiota that we have characterized in this study.

## Supplementary Material

fiaf022_Supplemental_Files Code for analyses is available at https://github.com/valbarberoux/R-script—Meuse-BCC

## References

[bib1] Abiriga D, Jenkins A, Alfsnes K et al. Characterisation of the bacterial microbiota of a landfill-contaminated confined aquifer undergoing intrinsic remediation. Sci Total Environ. 2021;785:147349. 10.1016/j.scitotenv.2021.147349.

[bib2] Adyasari D, Hassenrück C, Montiel D et al. Microbial community composition across a coastal hydrological system affected by submarine groundwater discharge (SGD). PLoS One. 2020;15:e0235235. 10.1371/journal.pone.0235235. 32598345 PMC7323985

[bib3] Aitchison J . The Statistical Analysis of Compositional Data. London: Chapman and Hall, 1986.

[bib4] Apprill A, McNally S, Parsons R et al. Minor revision to V4 region SSU rRNA 806R gene primer greatly increases detection of SAR11 bacterioplankton. Aquat Microb Ecol. 2015;75:129–37. 10.3354/ame01753.

[bib5] Atamna-Ismaeel N, Sabehi G, Sharon I et al. Widespread distribution of proteorhodopsins in freshwater and brackish ecosystems. ISME J. 2008;2:656–62. 10.1038/ismej.2008.27. 18369329

[bib6] Aylagas E, Borja Á, Tangherlini M et al. A bacterial community-based index to assess the ecological status of estuarine and coastal environments. Mar Pollut Bull. 2017;114:679–88. 10.1016/j.marpolbul.2016.10.050. 27784536

[bib160_920_055225] Azam F, Fenchel T, Field J et al. The Ecological Role of Water-Column Microbes in the Sea. Mar Ecol Prog Ser. 1983;10:257–63. 10.3354/meps010257

[bib7] Basiry D, Kommedal R, Kaster KM. The presence of antibiotic-resistant bacteria at four Norwegian wastewater treatment plants: seasonal and wastewater-source effects. Front Antibiot. 2024;3:1351999. 10.3389/frabi.2024.1351999. 39816252 PMC11731629

[bib8] Bian R, Huang S, Cao X et al. Spatial and temporal distribution of the microbial community structure in the receiving rivers of the middle and lower reaches of the Yangtze River under the influence of different wastewater types. J Hazard Mater. 2024;462:132835. 10.1016/j.jhazmat.2023.132835. 37879279

[bib9] Blackall LL, Hayward AC, Sly LI. Cellulolytic and dextranolytic Gram-negative bacteria: revival of the genus *Cellvibrio*. J Appl Bacteriol. 1985;59:81–97. 10.1111/j.1365-2672.1985.tb01779.x.

[bib10] Blais M-A, Matveev A, Lovejoy C et al. Size-fractionated microbiome structure in subArctic rivers and a coastal plume across DOC and salinity gradients. Front Microbiol. 2022;12:760282. 10.3389/fmicb.2021.760282. 35046910 PMC8762315

[bib11] Borges AV . Effects of agricultural land use on fluvial carbon dioxide, methane and nitrous oxide concentrations in a large European river, the Meuse (Belgium). Sci Total Environ. 2018:14. 10.1016/j.scitotenv.2017.08.047. 28806551

[bib12] Burkert U, Warnecke F, Babenzien D. et al. Members of a readily enriched β-proteobacterial clade are common in surface waters of a humic lake. Appl Environ Microbiol. 2003;69:6550–9. 10.1128/AEM.69.11.6550-6559.2003. 14602613 PMC262289

[bib13] Cadotte MW . Dispersal and species diversity: a meta-analysis. Am Nat. 2006;167:913–24. 10.1086/504850. 16649154

[bib14] Caillon F, Besemer K, Peduzzi P et al. Soil microbial inoculation during flood events shapes headwater stream microbial communities and diversity. Microb Ecol. 2021;82. 10.1007/s00248-021-01700-3. PMC8463373 33532913

[bib15] Calderón-Franco D, Sarelse R, Christou S et al. Metagenomic profiling and transfer dynamics of antibiotic resistance determinants in a full-scale granular sludge wastewater treatment plant. Water Res. 2022;219:118571. 10.1016/j.watres.2022.118571. 35576763

[bib16] Callahan BJ, Sankaran K, Fukuyama JA et al. Bioconductor workflow for microbiome data analysis: from raw reads to community analyses. F1000Res. 2016;5:1492. 10.12688/f1000research.8986.1. 27508062 PMC4955027

[bib17] Caporaso JG, Lauber CL, Walters WA et al. Global patterns of 16S rRNA diversity at a depth of millions of sequences per sample. Proc Natl Acad Sci USA. 2011;108:4516–22. 10.1038/ismej.2012.8. 20534432 PMC3063599

[bib18] Caporaso JG, Lauber CL, Walters WA et al. Ultra-high-throughput microbial community analysis on the Illumina HiSeq and MiSeq platforms. ISME J. 2012;6:1621–4. 10.1073/pnas.1000080107. 22402401 PMC3400413

[bib19] Carlson CA, Morris R, Parsons R et al. Seasonal dynamics of SAR11 populations in the euphotic and mesopelagic zones of the northwestern Sargasso Sea. ISME J. 2009;3:283–95. 10.1038/ismej.2008.117. 19052630

[bib21] Chen W-M, Liu L-P, Sheu S-Y. *Cellvibrio fontiphilus* sp. nov., isolated from a spring. Int J Syst Evol Microbiol. 2017;67:2532–7. 10.1099/ijsem.0.001952. 28771122

[bib20] Chen W, Gao S, Chang K et al. Partial fishmeal replacement by soybean meal induces fish growth retardation and gut inflammation via gut mucosal barrier dysfunction and dysbiosis in largemouth bass. Anim Feed Sci Technol. 2024;316:116067. 10.1016/j.anifeedsci.2024.116067.

[bib22] Cheng SM, Foght JM. Cultivation-independent and -dependent characterization of bacteria resident beneath John Evans Glacier: characterization of subglacial bacterial communities. FEMS Microbiol Ecol. 2007;59:318–30. 10.1111/j.1574-6941.2006.00267.x. 17313581

[bib23] Chiriac M, Haber M, Salcher MM. Adaptive genetic traits in pelagic freshwater microbes. Environ Microbiol. 2023;25:606–41. 10.1111/1462-2920.16313. 36513610

[bib25] Coclet C, Garnier C, Durrieu G et al. Changes in bacterioplankton communities resulting from direct and indirect interactions with trace metal gradients in an urbanized marine coastal area. Front Microbiol. 2019;10:257. 10.3389/fmicb.2019.00257. 30853948 PMC6395402

[bib26] Cruaud P, Vigneron A, Fradette M et al. Annual bacterial community cycle in a seasonally ice-covered river reflects environmental and climatic conditions. Limnol Oceanogr. 2020;65. 10.1002/lno.11130.

[bib27] Crump BC, Amaral-Zettler LA, Kling GW. Microbial diversity in arctic freshwaters is structured by inoculation of microbes from soils. ISME J. 2012;6:1629–39. 10.1038/ismej.2012.9. 22378536 PMC3498914

[bib28] Crump BC, Armbrust EV, Baross JA. Phylogenetic analysis of particle-attached and free-living bacterial communities in the Columbia River, its estuary, and the adjacent Coastal Ocean. Appl Environ Microbiol. 1999;65:3192–204. 10.1128/AEM.65.7.3192-3204. 10388721 PMC91474

[bib29] Crump BC, Baross J, Simenstad C. Dominance of particle-attached bacteria in the Columbia River estuary, USA. Aquat Microb Ecol. 1998;14:7–18. 10.1038/ismej.2012.9[.CrossRef]

[bib165_819_051925] Crump BC, Hobbie JE. Synchrony and seasonality in bacterioplankton communities of two temperate rivers. Limnol Oceanogr. 2005;50:1718–29. 10.4319/lo.2005.50.6.1718

[bib30] Crump BC, Hobbie JE. Synchrony and seasonality in bacterioplankton communities of two temperate rivers. Limnol Oceanogr. 2005;50:1718–29. 10.4319/lo.2005.50.6.1718.

[bib31] Crump BC, Peterson BJ, Raymond PA et al. Circumpolar synchrony in big river bacterioplankton. Proc Natl Acad Sci USA. 2009;106:21208–12. 10.1073/pnas.0906149106. 19940248 PMC2783008

[bib32] Davis TW, Berry DL, Boyer GL et al. The effects of temperature and nutrients on the growth and dynamics of toxic and non-toxic strains of microcystis during cyanobacteriota blooms. Harmful Algae. 2009;8:715–25. 10.1016/j.hal.2009.02.004.

[bib33] de Oliveira LFV, Margis R. The source of the river as a nursery for microbial diversity. PLoS One. 2015;10:e0120608. 10.1371/journal.pone.0120608. 25803426 PMC4372583

[bib34] Descy J-P, Gosselain V. Development and ecological importance of phytoplankton in a large lowland river (River Meuse, Belgium). Hydrobiologia. 1994;289:139–55. 10.1007/BF00007415.

[bib35] Descy J-P, Servais P, Smitz JS et al. Phytoplankton biomass and production in the River Meuse (Belgium). Water Res. 1987;21:1557–66. 10.1016/0043-1354(87)90141-2.

[bib37] Descy J-P . Continental Atlantic rivers. Rivers of Europe. Amsterdam: Elsevier, 2009,151–98. 10.1016/B978-0-12-369449-2.00005-9.

[bib36] Descy J-P . Phytoplankton production, exudation and bacterial reassimilation in the River Meuse (Belgium). J Plankton Res. 2002;24:161–6. 10.1093/plankt/24.3.161.

[bib38] Doherty M, Yager PL, Moran MA et al. Bacterial biogeography across the Amazon River-ocean continuum. Front Microbiol. 2017;8. 10.3389/fmicb.2017.00882. PMC5440517 28588561

[bib39] Eiler A, Hayakawa DH, Church MJ et al. Dynamics of the SAR11 bacterioplankton lineage in relation to environmental conditions in the oligotrophic North Pacific subtropical gyre. Environ Microbiol. 2009;11:2291–300. 10.1111/j.1462-2920.2009.01954.x. 19490029

[bib40] Eraqi WA, ElRakaiby MT, Megahed SA et al. Spatiotemporal analysis of the water and sediment Nile microbial community along an urban metropolis. Microb Ecol. 2021;82:288–98. 10.1007/s00248-020-01674-8. 33420624

[bib41] Farkas M, Kaszab E, Radó J et al. Planktonic and benthic bacterial communities of the largest Central European Shallow Lake, Lake Balaton and its main inflow Zala River. Curr Microbiol. 2020;77:4016–28. 10.1007/s00284-020-02241-7. 33068137 PMC7677278

[bib159_619_053925] Farkas M, Szoboszlay S, Vörös L et al. Bacterial Community Dynamics along a River-Wetland-Lake System. Water. 2022;14:3519. 10.3390/w14213519.

[bib42] Feng L-J, Xu J, Xu X-Y et al. Enhanced biological nitrogen removal via dissolved oxygen partitioning and step feeding in a simulated river bioreactor for contaminated source water remediation. Int Biodeterior Biodegrad. 2012;71:72–9. 10.1016/j.ibiod.2011.12.016.

[bib43] Fierer N, Morse JL, Berthrong ST et al. Environmental controls on the landscape-scale biogeography of stream bacterial communities. Ecology. 2007;88:2162–73. 10.1890/06-1746.1. 17918395

[bib44] Fontaine L, Pin L, Savio D et al. Bacterial bioindicators enable biological status classification along the continental Danube river. Commun Biol. 2023;6:862. 10.1038/s42003-023-05237-8. 37596339 PMC10439154

[bib45] Fukushima H, Gomyoda M, Shiozawa K et al. *Yersinia pseudotuberculosis* infection contracted through water contaminated by a wild animal. J Clin Microbiol. 1988;26:584–5. 10.1128/jcm.26.3.584-585.1988. 2833532 PMC266339

[bib46] Gloor GB, Macklaim JM, Pawlowsky-Glahn V et al. Microbiome datasets are compositional: and this is not optional. Front Microbiol. 2017;8:2224. 10.3389/fmicb.2017.02224. 29187837 PMC5695134

[bib47] Grossart HP . Ecological consequences of bacterioplankton lifestyles: changes in concepts are needed. Environ Microbiol. 2010;2:706–14. 10.1111/j.1758-2229.2010.00179.x[.CrossRef] 23766274

[bib162_583_055925] Guo D, Liang J, Chen W et al. Bacterial Community Analysis of Two NeighboringFreshwater Lakes Originating from One Lake. Pol J Environ Stud. 2020;30:111–7. 10.15244/pjoes/119094

[bib48] Gweon HS, Bowes MJ, Moorhouse HL et al. Contrasting community assembly processes structure lotic bacteria metacommunities along the river continuum. Environ Microbiol. 2021;23:484–98. 10.1111/1462-2920.15337. 33258525 PMC7898806

[bib49] Hagberg A, Gupta S, Rzhepishevska O et al. Do environmental pharmaceuticals affect the composition of bacterial communities in a freshwater stream? A case study of the Knivsta river in the south of Sweden. Sci Total Environ. 2021;763:142991. 10.1016/j.scitotenv.2020.142991. 33121787

[bib50] Hamers T, Kamstra JH, van Gils J et al. The influence of extreme river discharge conditions on the quality of suspended particulate matter in Rivers Meuse and Rhine (the Netherlands). Environ Res. 2015;143:241–55. 10.1016/j.envres.2015.10.019. 26519830

[bib51] Harke MJ, Gobler CJ. Global transcriptional responses of the toxic cyanobacterium, *Microcystis aeruginosa*, to nitrogen stress, phosphorus stress, and growth on organic matter. PLoS One. 2013;8:e69834. 10.1371/journal.pone.0069834. 23894552 PMC3720943

[bib52] Hassell N, Tinker KA, Moore T et al. Temporal and spatial dynamics in microbial community composition within a temperate stream network: microbial community assembly in streams. Environ Microbiol. 2018;20:3560–72. 10.1111/1462-2920.14311. 30051569

[bib53] Heinrich F, Eiler A, Bertilsson S. Seasonality and environmental control of freshwater SAR11 (LD12) in a temperate lake (Lake Erken, Sweden). Aquat Microb Ecol. 2013;70:33–44. 10.3354/ame01637.

[bib54] Henson MW, Hanssen J, Spooner G et al. Nutrient dynamics and stream order influence microbial community patterns along a 2914 kilometer transect of the Mississippi River. Limnol Oceanogr. 2018;63:1837–55. 10.1002/lno.10811.

[bib55] Hosen JD, Febria CM, Crump BC et al. Watershed urbanization linked to differences in stream bacterial community composition. Front Microbiol. 2017;8. 10.3389/fmicb.2017.01452. PMC5539594 28824582

[bib56] Hu A, Ju F, Hou L et al. Strong impact of anthropogenic contamination on the co-occurrence patterns of a riverine microbial community: co-occurrence network of riverine microbiome. Environ Microbiol. 2017;19:4993–5009. 10.1111/1462-2920.13942. 28967165

[bib57] Hu M, Wang X, Wen X et al. Microbial community structures in different wastewater treatment plants as revealed by 454-pyrosequencing analysis. Bioresour Technol. 2012;117:72–9. 10.1016/j.biortech.2012.04.061. 22609716

[bib58] Hu Y, Bai C, Cai J et al. Co-occurrence network reveals the higher fragmentation of the bacterial community in Kaidu River than its tributaries in Northwestern China. Microbes Environ. 2018;33:127–34. 10.1264/jsme2.ME17170. 29794413 PMC6031398

[bib59] Hu Y, Xie G, Jiang X et al. The relationships between the free-living and particle-attached bacterial communities in response to elevated eutrophication. Front Microbiol. 2020;11:423. 10.3389/fmicb.2020.00423. 32269552 PMC7109266

[bib60] Huang S, Chen C, Jaffé PR. Seasonal distribution of nitrifiers and denitrifiers in urban river sediments affected by agricultural activities. Sci Total Environ. 2018;642:1282–91. 10.1016/j.scitotenv.2018.06.116. 30045508

[bib62] Jackson CR, Millar JJ, Payne JT et al. Free-living and particle-associated bacterioplankton in large rivers of the Mississippi River basin demonstrate biogeographic patterns. Appl Environ Microbiol. 2014;80:7186–95. 10.1128/AEM.01844-14. 25217018 PMC4249191

[bib63] Jezbera J, Jezberová J, Kasalický V et al. Patterns of *Limnohabitans* microdiversity across a large set of freshwater habitats as revealed by reverse line blot hybridization. PLoS One. 2013;8:e58527. 10.1371/journal.pone.0058527. 23554898 PMC3595293

[bib64] Jezberová J, Komárková J. Morphological transformation in a freshwater *Cyanobium* sp. induced by grazers. Environ Microbiol. 2007;9:1858–62. 10.1111/j.1462-2920.2007.01311.x. 17564619

[bib65] Ji B, Qin H, Guo S et al. Bacterial communities of four adjacent fresh lakes at different trophic status. Ecotoxicol Environ Saf. 2018;157:388–94. 10.1016/j.ecoenv.2018.03.086. 29649784

[bib66] Ji L, Zhang L, Wang Z et al. High biodiversity and distinct assembly patterns of microbial communities in groundwater compared with surface water. Sci Total Environ. 2022;834:0048–9697. 10.1016/j.scitotenv.2022.155345. 35460778

[bib67] Jin H, Li L, Lu W et al. Identification of the regulatory roles of water qualities on the spatio–temporal dynamics of microbiota communities in the water and fish guts in the Heilongjiang River. Front Microbiol. 2024;15:1435360. 10.3389/fmicb.2024.1435360. 39234540 PMC11372393

[bib68] Joaquim-Justo C, Pirlot S, Viroux L et al. Trophic links in the lowland River Meuse (Belgium): assessing the role of bacteria and protozoans in planktonic food webs. J Plankton Res. 2006;28:857–70. 10.1093/plankt/fbl021.

[bib69] Jordaan K, Bezuidenhout C. The impact of physico-chemical water quality parameters on bacterial diversity in the Vaal River, South Africa. WSA. 2013;39:385–96. 10.4314/wsa.v39i3.7.

[bib70] Judd KE, Crump BC, Kling GW. Variaiton in dossolved organic matter controls bacterial production and community composition. Ecology. 2006;87:2068–79. 10.1890/0012-9658(2006)87[2068:VIDOMC]2.0.CO;2. 16937646

[bib71] Kaden R, Spröer C, Beyer D et al. *Rhodoferax saidenbachensis* sp. nov., a psychrotolerant, very slowly growing bacterium within the family Comamonadaceae, proposal of appropriate taxonomic position of *Albidiferax ferrireducens* strain T118T in the genus *Rhodoferax* and emended description of the genus *Rhodoferax*. Int J Syst Evol Microbiol. 2014;64:1186–93. 10.1099/ijs.0.054031-0. 24408525

[bib164_921_051525] Kämpfer P, Busse H-J, Longaric I et al. Pseudarcicella hirudinis gen. nov., sp. nov., isolated from the skin of the medical leech Hirudo medicinalis. Int J Syst Evol Microbiol. 2012;62:2247–51. 10.1099/ijs.0.037390-0 22081719

[bib72] Kang I, Kim S, Islam MR et al. The first complete genome sequences of the acI lineage, the most abundant freshwater actinobacteria, obtained by whole-genome-amplification of dilution-to-extinction cultures. Sci Rep. 2017;7:42252. 10.1038/srep42252. 28186143 PMC5301498

[bib73] Kasalický V, Jezbera J, Hahn MW et al. The diversity of the *Limnohabitans* genus, an important group of freshwater bacterioplankton, by characterization of 35 isolated strains. PLoS One. 2013;8:e58209. 10.1371/journal.pone.0058209. 23505469 PMC3591437

[bib74] Kasanke CP, Willis MD, Leigh MB. Distribution of a sulfolane-metabolizing *Rhodoferax* sp. throughout a contaminated subarctic aquifer and two groundwater treatment systems. Front Microbiol. 2021;12:714769. 10.3389/fmicb.2021.714769. 34512592 PMC8427821

[bib76] Kirchman DL . The ecology of cytophaga–flavobacteria in aquatic environments. FEMS Microbiol Ecol. 2002;39:91–100. 10.1111/j.1574-6941.2002.tb00910.x. 19709188

[bib77] Kolda A, Ljubešić Z, Gavrilović A et al. Metabarcoding cyanobacteriota in coastal waters and sediment in central and southern Adriatic Sea. Acta Bot Croat. 2020;79:157–69. 10.37427/botcro-2020-021.

[bib78] Kumar R, Verma H, Haider S et al. Comparative genomic analysis reveals habitat-specific genes and regulatory hubs within the genus *Novosphingobium*. mSystems. 2017;2:e00020–17. 10.1128/mSystems.00020-17. 28567447 PMC5443232

[bib79] Laperriere SM, Hilderbrand RH, Keller SR et al. Headwater stream microbial diversity and function across agricultural and urban land use gradients. Appl Environ Microbiol. 2020;86:e00018–20. 10.1128/AEM.00018-20. 32245755 PMC7237783

[bib80] Lau N-S, Furusawa G. Polysaccharide degradation in cellvibrionaceae: genomic insights of the novel chitin-degrading marine bacterium, strain KSP-S5-2, and its chitinolytic activity. Sci Total Environ. 2024;912:169134. 10.1016/j.scitotenv.2023.169134. 38070563

[bib81] Lee J-C, Kim S-G, Whang K-S. *Novosphingobium aquiterrae* sp. nov., isolated from ground water. Int J Syst Evol Microbiol. 2014;64:3282–7. 10.1099/ijs.0.060749-0. 24994774

[bib82] Leibold MA, Chase JM, Ernest SKM. Community assembly and the functioning of ecosystems: how metacommunity processes alter ecosystems attributes. Ecology. 2017;98:909–19. 10.1002/ecy.1697. 27984663

[bib83] Li Y, Zhao L, Niu L et al. Effect of pressure treatment on microcystis blooms and the subsequent succession of bacterial community. Algal Res. 2023;71:103023. 10.1016/j.algal.2023.103023.

[bib84] Liu K, Hou J, Liu Y et al. Biogeography of the free-living and particle-attached bacteria in Tibetan lakes. FEMS Microbiol Ecol. 2019;95:fiz088. 10.1093/femsec/fiz088. 31183497

[bib85] Liu T, Zhang AN, Wang J et al. Integrated biogeography of planktonic and sedimentary bacterial communities in the Yangtze River. Microbiome. 2018;6:16. 10.1186/s40168-017-0388-x. 29351813 PMC5775685

[bib86] Livermore JA, Emrich SJ, Tan J et al. Freshwater bacterial lifestyles inferred from comparative genomics: diverse freshwater bacteria genomes. Environ Microbiol. 2014;16:746–58. 10.1111/1462-2920.12199. 23889754

[bib87] Luef B, Aspetsberger F, Hein T et al. Impact of hydrology on free-living and particle-associated microorganisms in a river floodplain system (Danube, Austria). Freshwater Biol. 2007;52:1043–57. 10.1111/j.1365-2427.2007.01752.x.

[bib88] Ma L, Mao G, Liu J et al. Spatial-temporal changes of bacterioplankton community along an Exhorheic River. Front Microbiol. 2016;7. 10.3389/fmicb.2016.00250. PMC4776164 26973627

[bib89] Marescaux J, Boets P, Lorquet J et al. Sympatric *Dreissena* species in the Meuse River: towards a dominance shift from zebra to quagga mussels. AI. 2015;10:287–98. 10.3391/ai.2015.10.3.04.

[bib90] Martínez-Santos M, Lanzén A, Unda-Calvo J et al. Treated and untreated wastewater effluents alter river sediment bacterial communities involved in nitrogen and sulphur cycling. Sci Total Environ. 2018;633:1051–61. 10.1016/j.scitotenv.2018.03.229. 29758858

[bib91] Mateus-Barros E, de Melo ML, Bagatini IL et al. Local and geographic factors shape the occupancy-frequency distribution of freshwater bacteria. Microb Ecol. 2021;81:26–35. 10.1007/s00248-020-01560-3. 32705311

[bib92] Mergaert J, Lednická D, Goris J et al. Taxonomic study of *Cellvibrio* strains and description of *Cellvibrio ostraviensis* sp. nov., *Cellvibrio fibrivorans* sp. nov. and *Cellvibrio gandavensis* sp. nov. Int J Syst Evol Microbiol. 2003;53:465–71. 10.1099/ijs.0.02316-0[. 12710614

[bib93] Millar EN, Kidd KA, Surette MG et al. Effects of municipal wastewater effluents on the digestive gland microbiome of wild freshwater mussels (*Lasmigona costata*). Ecotoxicol Environ Saf. 2022;241:113774. 10.1016/j.ecoenv.2022.113774. 35777341

[bib94] Mohit V, Archambault P, Toupoint N et al. Phylogenetic differences in attached and free-living bacterial communities in a temperate coastal lagoon during summer, revealed via high-throughput 16S rRNA gene sequencing. Appl Environ Microbiol. 2014;80:2071–83. 10.1128/AEM.02916-13. 24463966 PMC3993158

[bib95] Mouquet N, Loreau M. Coexistence in metacommunities: the regional similarity hypothesis. Am Nat. 2002;159:420–6. 10.1086/338996. 18707425

[bib96] Mouquet N, Loreau M. Community patterns in source-sink metacommunities. Am Nat. 2003;162:544–57. 10.1086/378857. 14618534

[bib97] Nakai R, Baba T, Niki H et al. *Aurantimicrobium minutum* gen. nov., sp. nov., a novel ultramicrobacterium of the family Microbacteriaceae, isolated from river water. Int J Syst Evol Microbiol. 2015;65:4072–9. 10.1099/ijsem.0.000541. 26294911

[bib98] Newton RJ, Jones SE, Helmus MR et al. Phylogenetic ecology of the freshwater *Actinobacteria* acI lineage. Appl Environ Microbiol. 2007;75:7169–76. 10.1128/AEM.00794-07. PMC2168227 17827330

[bib99] Niño-García JP, Ruiz-González C, del Giorgio PA. Interactions between hydrology and water chemistry shape bacterioplankton biogeography across boreal freshwater networks. ISME J. 2016;10:1755–66. 10.1038/ismej.2015.226. 26849312 PMC4918434

[bib100] Okafor N . Ecology of microorganisms in freshwater. In: Environmental Microbiology of Aquatic and Waste Systems. Dordrecht: Springer, 2011,111–22. 10.1007/978-94-007-1460-1_5.

[bib101] Oksanen J, Blanchet F, Kindt R et al. Package ‘vegan’: community ecology package. Version 2.6-10. CRAN, 2019, 1–295. Retrieved March 8, 2021, from https://github.com/vegandevs/vegan.

[bib102] Ortega-Retuerta E, Joux F, Jeffrey WH et al. Spatial variability of particle-attached and free-living bacterial diversity in surface waters from the Mackenzie River to the Beaufort Sea (Canadian Arctic). Biogeosciences. 2013;10:2747–59. 10.5194/bg-10-2747-2013.

[bib103] Pandey PK, Kass PH, Soupir ML et al. Contamination of water resources by pathogenic bacteria. AMB Expr. 2014;4:51. 10.1186/s13568-014-0051-x. PMC4077002 25006540

[bib104] Parada AE, Needham DM, Fuhrman JA. Every base matters: assessing small subunit rRNA primers for marine microbiomes with mock communities, time series and global field samples: primers for marine microbiome studies. Environ Microbiol. 2016;18:1403–14. 10.1111/1462-2920.13023. 26271760

[bib105] Paudel Adhikari N, Liu Y, Liu K et al. Bacterial community composition and diversity in Koshi River, the largest river of Nepal. Ecol Indic. 2019;104:501–11. 10.1016/j.ecolind.2019.05.009.

[bib106] Payne JT, Jackson CR, Millar JJ et al. Timescales of variation in diversity and production of bacterioplankton assemblages in the Lower Mississippi River. PLoS One. 2020;15:e0230945. 10.1371/journal.pone.0230945. 32255790 PMC7138331

[bib107] Payne JT, Millar JJ, Jackson CR et al. Patterns of variation in diversity of the Mississippi River microbiome over 1300 kilometers. PLoS One. 2017;12:e0174890. 10.1371/journal.pone.0174890. 28350888 PMC5370145

[bib108] Pernthaler J . Competition and niche separation of pelagic bacteria in freshwater habitats: niches of freshwater bacterioplankton. Environ Microbiol. 2017;19:2133–50. 10.1111/1462-2920.13742. 28370850

[bib109] Pigneur L-M, Falisse E, Roland K et al. Impact of invasive Asian clams, *Corbicula* spp., on a large river ecosystem. Freshw Biol. 2014;59:573–83. 10.1111/fwb.12286.

[bib110] Pinto CT, Nano FE. Stable, temperature-sensitive recombinant strain of *Mycobacterium smegmatis* generated through the substitution of a psychrophilic *ligA* gene. FEMS Microbiol Lett. 2015;362:fnv152. 10.1093/femsle/fnv152. 26337150

[bib111] Pitt A, Koll U, Schmidt J et al. *Aquirufa lenticrescens* sp. nov. and *Aquirufa aurantiipilula* sp. nov.: two new species of a lineage of widespread freshwater bacteria. Arch Microbiol. 2022;204:356. 10.1007/s00203-022-02950-6. 35654990 PMC9163014

[bib112] Pitt A, Schmidt J, Koll U et al. *Aquirufa antheringensis* gen. nov., sp. nov. and *Aquirufa nivalisilvae* sp. nov., representing a new genus of widespread freshwater bacteria. Int J Syst Evol Microbiol. 2019;69:2739–49. 10.1099/ijsem.0.003554. 31259682

[bib113] R Studio Team . RStudio: integrated development for R. Boston: PBC, 2020. Retrieved February 4, 2020, from http://www.rstudio.com/.

[bib114] Read DS, Gweon HS, Bowes MJ et al. Catchment-scale biogeography of riverine bacterioplankton. ISME J. 2015;9:516–26. 10.1038/ismej.2014.166. 25238398 PMC4303643

[bib115] Retter A, Haas JC, Birk S et al. From the mountain to the valley: drivers of groundwater prokaryotic communities along an Alpine River corridor. Microorganisms. 2023;11:779. 10.3390/microorganisms11030779. 36985351 PMC10055094

[bib116] Reza MS, Mizusawa N, Kumano A et al. Metagenomic analysis using 16S ribosomal RNA genes of a bacterial community in an urban stream, the Tama River, Tokyo. Fish Sci. 2018;84:563–77. 10.1007/s12562-018-1193-6.

[bib117] Rieck A, Herlemann DPR, Jürgens K et al. Particle-associated differ from free-living bacteria in surface waters of the Baltic Sea. Front Microbiol. 2015;6. 10.3389/fmicb.2015.01297. PMC4664634 26648911

[bib118] Ruiz-González C, Niño-García JP, Lapierre J-F et al. The quality of organic matter shapes the functional biogeography of bacterioplankton across boreal freshwater ecosystems: the functional biogeography of bacteria. Global Ecol Biogeogr. 2015;24:1487–98. 10.1111/geb.12356.

[bib119] Salcher MM, Ewert C, Šimek K et al. Interspecific competition and protistan grazing affect the coexistence of freshwater betaproteobacterial strains. FEMS Microbiol Ecol. 2015;92:fiv156. 10.1093/femsec/fiv156. 26656063

[bib120] Salcher MM, Pernthaler J, Posch T. Seasonal bloom dynamics and ecophysiology of the freshwater sister clade of SAR11 bacteria ‘that rule the waves’ (LD12). ISME J. 2011;5:1242–52. 10.1038/ismej.2011.8. 21412347 PMC3146277

[bib121] Saunders AM, Albertsen M, Vollertsen J et al. The activated sludge ecosystem contains a core community of abundant organisms. ISME J. 2016;10:11–20. 10.1038/ismej.2015.117. 26262816 PMC4681854

[bib122] Savio D, Sinclair L, Ijaz UZ et al. Bacterial diversity along a 2600 km river continuum: river bacterioplankton diversity. Environ Microbiol. 2015;17:4994–5007. 10.1111/1462-2920.12886. 25922985 PMC4918796

[bib123] Servais P, Gosselain V, Joaquim-Justo C et al. Trophic relationships between planktonic microorganisms in the river Meuse (Belgium): a carbon budget. Archiv Für Hydrobiologie. 2000;149:625–53. 10.1127/archiv-hydrobiol/149/2000/625.

[bib124] Servais P . Bacterioplanktonic biomass and production in the river Meuse (Belgium). Hydrobiologia. 1989;174:99–110. 10.1007/BF00014058.

[bib125] Shanafelt DW, Dieckmann U, Jonas M et al. Biodiversity, productivity, and the spatial insurance hypothesis revisited. J Theor Biol. 2015;380:426–35. 10.1016/j.jtbi.2015.06.017. 26100182 PMC4559352

[bib126] Sheu S-Y, Chen T-Y, Chen W-M. *Aquirufa rosea* sp. nov., isolated from a freshwater lake. Int J Syst Evol Microbiol. 2020;70:3145–53. 10.1099/ijsem.0.004147. 32267219

[bib127] Sheu S-Y, Liu L-P, Chen W-M. *Novosphingobium bradum* sp. nov., isolated from a spring. Int J Syst Evol Microbiol. 2016;66:5083–90. 10.1099/ijsem.0.001475. 27599476

[bib128] Šimek K, Kasalický V, Zapomělová E et al. Alga-derived substrates select for distinct betaproteobacterial lineages and contribute to niche separation in limnohabitans strains. Appl Environ Microbiol. 2011;77:7307–15. 10.1128/AEM.05107-11. 21873481 PMC3194872

[bib129] Sommaruga R, Casamayor EO. Bacterial ‘cosmopolitanism’ and importance of local environmental factors for community composition in remote high-altitude lakes. Freshwater Biol. 2009;54:994–1005. 10.1111/j.1365-2427.2008.02146.x. PMC2883735 20543908

[bib130] Song Y, Jia J, Liu D et al. *Sediminibacterium roseum* sp. nov., isolated from sewage sediment. Int J Syst Evol Microbiol. 2017;67:4674–9. 10.1099/ijsem.0.002355. 28984225

[bib131] Spietz RL, Williams CM, Rocap G et al. A dissolved oxygen threshold for shifts in bacterial community structure in a seasonally hypoxic estuary. PLoS One. 2015;10:e0135731. 10.1371/journal.pone.0135731. 26270047 PMC4535773

[bib132] Staley C, Gould TJ, Wang P et al. Core functional traits of bacterial communities in the Upper Mississippi River show limited variation in response to land cover. Front Microbiol. 2014;5. 10.3389/fmicb.2014.00414. PMC4126211 25152748

[bib133] Staley C, Unno T, Gould TJ et al. Application of Illumina next-generation sequencing to characterize the bacterial community of the Upper Mississippi River. J Appl Microbiol. 2013;115:1147–58. 10.1111/jam.12323. 23924231

[bib134] Stanier R, Kunisawa R, Mandel M et al. Purification and properties of unicellular bluegreen algae (order Chroococcales). Bacteriol Rev. 1971;35:171. 10.1128/br.35.2.171-205.1971. 4998365 PMC378380

[bib135] Steven B, Pollard WH, Greer CW et al. Microbial diversity and activity through a permafrost/ground ice core profile from the Canadian high Arctic. Environ Microbiol. 2008;10:3388–403. 10.1111/j.1462-2920.2008.01746.x. 19025556

[bib136] Sulakvelidze A . *Yersiniae* other than *Y. enterocolitica, Y. pseudotuberculosis*, and *Y. pestis*: the ignored species. Microbes Infect. 2000;2. 10.1016/s1286-4579(00)00311-7. 10865195

[bib137] Sun X, Su L, Zhen J et al. The contribution of swine wastewater on environmental pathogens and antibiotic resistance genes: antibiotic residues and beyond. Chemosphere. 2024;364:143263. 10.1016/j.chemosphere.2024.143263. 39236924

[bib138] Sun Y, Wang S, Niu J. Microbial community evolution of black and stinking rivers during *in situ* remediation through micro-nano bubble and submerged resin floating bed technology. Bioresour Technol. 2018;258:187–94. 10.1016/j.biortech.2018.03.008. 29525593

[bib139] Suzuki Y, Economo EP. From species sorting to mass effects: spatial network structure mediates the shift between metacommunity archetypes. Ecography. 2021;44:715–26. 10.1111/ecog.05453.

[bib140] Tamaki H, Hanada S, Sekiguchi Y et al. Effect of gelling agent on colony formation in solid cultivation of microbial community in lake sediment. Environ Microbiol. 2009;11:1827–34. 10.1111/j.1462-2920.2009.01907.x. 19302540

[bib141] Teachey ME, McDonald JM, Ottesen EA. Rapid and stable microbial community assembly in the headwaters of a third-order stream. Appl Environ Microbiol. 2019;85:e00188–19. 10.1128/AEM.00188-19. 30952660 PMC6532045

[bib142] Thompson PL, Gonzalez A. Ecosystem multifunctionality in metacommunities. Ecology. 2016;97:2867–79. 10.1002/ecy.1502. 27859122

[bib143] Tsementzi D, Rodriguez-R LM, Ruiz-Perez CA et al. Ecogenomic characterization of widespread, closely-related SAR11 clades of the freshwater genus “*Candidatus Fonsibacter*” and proposal of Ca. *Fonsibacter lacus* sp. nov. Syst Appl Microbiol. 2019;42:495–505. 10.1016/j.syapm.2019.03.007. 31085022

[bib145] Van Rossum T, Peabody MA, Uyaguari-Diaz MI et al. Year-long metagenomic study of river microbiomes across land use and water quality. Front Microbiol. 2015;6. 10.3389/fmicb.2015.01405. PMC4681185 26733955

[bib146] Van Trappen S, Mergaert J, Van Eygen S et al. Diversity of 746 heterotrophic bacteria isolated from microbial mats from ten antarctic lakes. Syst Appl Microbiol. 2002;25:603–10. 10.1078/07232020260517742. 12583721

[bib144] Vannote RL, Minshall GW, Cummins KW et al. The River Continuum concept. Can J Fish Aquat Sci. 1980;37:130–7. 10.1139/f80-017.

[bib147] Velimirov B, Milosevic N, Kavka GG et al. Development of the bacterial compartment along the Danube River: a continuum despite local influences. Microb Ecol. 2011;61:955–67. 10.1007/s00248-010-9768-5. 21080161

[bib148] Vignale FA, Bernal Rey D, Pardo AM et al. Spatial and seasonal variations in the bacterial community of an anthropogenic impacted urban stream. Microb Ecol. 2023;85:862–74. 10.1139/f80-01710.1007/s00248-022-02055-z. 35701635

[bib149] Wang H, Liu X, Wang Y et al. Spatial and temporal dynamics of microbial community composition and factors influencing the surface water and sediments of urban rivers. J Environ Sci. 2023;124:187–97. 10.1016/j.jes.2021.10.016. 36182129

[bib150] Wang P, Chen B, Yuan R et al. Characteristics of aquatic bacterial community and the influencing factors in an urban river. Sci Total Environ. 2016;569–570:382–9. 10.1016/j.scitotenv.2016.06.130. 27348702

[bib151] Wang P, Zhao J, Xiao H et al. Bacterial community composition shaped by water chemistry and geographic distance in an anthropogenically disturbed river. Sci Total Environ. 2019;655:61–9. 10.1016/j.scitotenv.2018.11.234. 30469069

[bib152] Wang S, Dong RM, Dong CZ et al. Diversity of microbial plankton across the three Gorges Dam of the Yangtze River, China. Geoscience Front. 2012;3:335–49. 10.1016/j.gsf.2011.11.013.

[bib153] Winter C, Hein T, Kavka G et al. Longitudinal changes in the bacterial community composition of the Danube River: a whole-river approach. AEM. 2007;73: 421–31. 10.1128/AEM.01849-06. PMC1796958 17085708

[bib154] Wu G, Ge L, Zhao N et al. Environment dependent microbial co-occurrences across a cyanobacteriotal bloom in a freshwater lake. Environ Microbiol. 2021;23:327–39. 10.1111/1462-2920.15315. 33185973

[bib155] Xiao M, Li M, Reynolds CS. Colony formation in the *Cyanobacterium microcystis*. Biol Rev. 2018;93:1399–420. 10.1111/brv.12401. 29473286

[bib156] Xie Z, Lin W, Luo J. Comparative phenotype and genome analysis of *Cellvibrio* sp. PR1, a xylanolytic and agarolytic bacterium from the Pearl River. Biomed Res Int. 2017;2017:1–10. 10.1155/2017/6304248. PMC5536142 28798934

[bib157] Yang Y, Li S, Gao Y et al. Environment-driven geographical distribution of bacterial communities and identification of indicator taxa in Songhua River. Ecol Indic. 2019;101:62–70. 10.1016/j.ecolind.2018.12.047.

[bib158] Zhang W, Ki J-S, Qian P-Y. Microbial diversity in polluted harbor sediments I: bacterial community assessment based on four clone libraries of 16S rDNA. Estuar Coastal Shelf Sci. 2008;76:668–81. 10.1016/j.ecss.2007.07.040.

[bib159] Zhang Y, Xu J, Wang E et al. Mechanisms underlying the rhizosphere-to-rhizoplane enrichment of *Cellvibrio* unveiled by genome-centric metagenomics and metatranscriptomics. Microorganisms. 2020;8:583. 10.3390/microorganisms8040583. 32316533 PMC7232360

[bib160] Zhao Y, Wang X, Yang J et al. A modified slow sand filtration system of epikarst spring water in karst mountainous areas, China. J Water Health. 2021;19:229–41. 10.2166/wh.2021.242. 33901020

[bib161] Zhuang K, Izallalen M, Mouser P et al. Genome-scale dynamic modeling of the competition between *Rhodoferax* and *Geobacter* in anoxic subsurface environments. ISME J. 2011;5:305–16. 10.1038/ismej.2010.117. 20668487 PMC3105697

