# Supplementary material for: Spatio–temporal dynamics of bacterial community composition in a Western European watershed, the Meuse River watershed
Source: FEMS Microbiol Ecol. 2025 Mar 5;101(4):fiaf022. doi: 10.1093/femsec/fiaf022 (PMC11916896; doi:10.1093/femsec/fiaf022)
Supplement: fiaf022_Supplemental_Files — Code for analyses is available at https://github.com/valbarberoux/R-script—Meuse-BCC [file fiaf022_supplemental_files.zip › Supplementary material.docx]

**Supplementary material**

**Supplementary protocols**

**(A)biotic parameters measurements**

*Temperature and dissolved oxygen*

On site, temperature and dissolved oxygen were measured with a portable multiparametric probe (VWR DO210, VWR, USA).

*Bacterial production*

On site, 10 mL of water were poured into 50-mL bottles. These were stored in boxes filled with river water of the corresponding sampling sites in order to maintain a temperature as similar as possible to that of the sampling locations. At the laboratory, 100µL of tritiated thymidine (1.86 nM Thymidin methyl-^3^H, 5mCi 185MBq, PerkinElmer, USA) were added to 10mL of non-filtered water samples. Samples were incubated for one hour in the boxes previously cited. Afterwards, 5 mL TCA (trichloracetic acid) 15% were added to the samples to stop thymidine incorporation by the bacteria. Then, samples were filtrated on cellulose acetate membranes (0.2µm pore-sized, Whatman, Germany). Membranes were placed in small plastic flasks and 10mL of liquid scintillation cocktail (FILTER-COUNT^TM^, PerkinElmer, USA) was added. The radioactivity incorporated in the bacteria collected on the membrane was measured with a liquid scintillator (TRI-CARB 2100TR, Packard).

*Total suspended matter (TSM)*

River samples were filtered on 0.7µm pore-sized glass microfiber filters (GFF, Whatman, Germany) previously weighted and desiccated (at least for 24h before use). Filters were then placed in an oven at 70°C during 24h. Afterwards, they were weighted again to determine dry TSM.

*Chlorophyll a (Chl-a)*

Water samples were filtered on the same filters than for TSM. Membranes were soaked in 5mL of acetone 90% at 4°C during 12h to extract chlorophyll pigments. Afterwards, the tubes were centrifugated for 2min at 1000 rpm using a 3-16PK centrifuge (Sigma-Aldrich, Merck, USA) in order to remove particles. A first measure was performed to obtain the concentration of chlorophyll *a* and phaeopigments, then 100 µL of HCl 0.1M was added to degrade Chl-a into phaeopigments and a second measure was performed after 30s. The difference between those two values corresponds to the concentration of Chl-a. These concentrations were measured with a UV-visible spectrophotometer (Lambda 650S, PerkinElmer, USA). An intact filter poured in 5mL acetone was used as blank.

*Ammonium*

3mL of reactive one (made by dissolving 35g of phenol (C_6_H_5_OH) and 0.4g of sodium nitroprussiate (Na_2_(Fe(CN)_5_NO.2H_2_O) in 1L of Milli-Q water) and 3 mL of reactive two (made by dissolving 140g of trisodic citrate (C_6_H_5_Na_3_O_7_.2H_2_O), 11g of NaOH and 35 NaClO in 500 mL of purified water) were added to 30mL of water samples. Those were heated at 80°C in the dark during 10 min. After cooling to ambient temperature, absorbance was measured at 630 nm with the same spectrophotometer than for Chl-a.

*Phosphate*

2 mL of reagent (which was a mix of a solution of 100mL of ascorbic acid (0.31M), 50 mL of K(SbO)C_4_H_4_O_6_ • 0.5 H_2_O (4mM), 100mL of (NH_4_)_6_Mo_7_O_24_•4H_2_O (0.02M) and 250mL of diluted H_2_SO_4_ (37.8mL of H_2_SO_4_ (relative density of 1.84) in 250mL of Milli-Q water) were added to 20 mL of sample. After addition of the reagent, samples were kept in the dark for minimum 5 min and maximum 2 h so that the coloration could develop. Absorbance was measured with the same spectrophotometer than previously cited.

**DNA extraction**

After filtration, the Durapore filters were cut into small pieces. For each sample, three mL of TE buffer (10 mM tris, 1mM EDTA, pH 8) were added in a 15-mL tube with the fragments of filter. 50 mg/mL of lysozyme were added (Thermo Fischer Scientific, USA), in order to degrade the cell wall, as well as 1.5 unit/mL of mutanolysine (Sigma-Aldrich, Merck, USA). Tubes were then vortexed for 30 sec and incubated in a water bath at 37°C for 30 min. Then, 0.5 mL of SDS (Sodium Dodecyl Sulfate) 25% was added.

Afterwards, 120 μL of proteinase K (19.2 mg/mL) (Thermo Fischer Scientific, USA) were incorporated to remove proteins in the samples, and tubes were vortexed for one min, then incubated at 60°C in a water bath for 30 min. Then, 3 cycles of -80°C during 10 min / 65°C during 5 min were performed. After this step, samples were stored at -80°C until further extraction steps.

To separate DNA from other cell components, five mL of warm Phenol Chloroform Isoamyl alcohol (65°C) were added to each sample under the hood. Tubes were vortexed several times for 10s. Then, they were centrifugated for 5 min at 8000 rpm, in order to separate the liquid in three phases. The aqueous phase (upper phase) was collected and transferred into 1.5mL sterile Eppendorf tube.

For removing polysaccharides from DNA, 70 μL of Sodium acetate 3M were added. Indeed, Na^+^ at high concentration provokes high ionic forces which neutralize the phosphate groups of the DNA. Hence, DNA becomes less soluble due to reduced interactions with water, while polysaccharides remain in solution as their solubility is not significantly affected by the presence of sodium ions.

To recover DNA by precipitation, 490 μL of isopropanol were added, and the tubes were then left for minimum 15 min at -20°C to favor precipitation. Subsequently, they were centrifuged at 14000 rpm at 4°C during 30 min. The supernatant was discarded and the pellet of DNA was rinsed by resuspension in one mL of ethanol 70% in order to remove the remaining impurities. The mix was centrifugated at 14000 rpm, 4°C for 5 min and the supernatant (ethanol) was removed. The Eppendorf tubes were left open to dry for 15 min. 50 μL TE buffer were finally added to the tubes, and all tubes corresponding to the same sample were pooled into a single DNA extract. DNA concentration and purity (A260/A280 and A260/A230 ratios) were measured using a Nanodrop spectrophotometer (Thermo Fischer Scientific, USA).

**Legend of supplementary figures**

- **Supplementary Figure S1**: Discharge of the Meuse river– A. during the spatial studies of the MR in spring and summer; B. during the annual study at Jambes. The temporal sampling site (Jambes) is marked on Fig. S1A. Not all sample kms are present on the x-axis of Fig. 2B to keep it readable. Km 926 corresponds to the river source.
- **Supplementary Figure S2**: Spearman correlation matrix between (a)biotic parameters and the 20 most abundant genera (italic) in the Meuse river spatial study (HW excluded, both seasons and both fractions aggregated) The scale and therefore the dot sizes correspond to the value of the Spearman correlation coefficient (ρ). Only correlations with a p-value <0.05 are represented by a symbol.
- **Supplementary Figure S3**: RDA representing all samples (headwaters, spatial studies on the main river, and temporal study) colored by fraction size. (A)biotic parameters measured are presented as vectors. Temp=Temperature –TSM=Total Suspended Matter – DO=Dissolved Oxygen – COD=Chemical Oxygen Demand – Chlo-a=Chlorophyll a
